# Monkeypox virus: phylogenomics, host–pathogen interactome and mutational cascade

**DOI:** 10.1099/mgen.0.000987

**Published:** 2023-04-12

**Authors:** Roshan Kumar, Shekhar Nagar, Shazia Haider, Utkarsh Sood, Kalaiarasan Ponnusamy, Gauri Garg Dhingra, Shailly Anand, Ankita Dua, Mona Singh, Roushan Kumar, Manisha Sengar, Indrakant Kumar Singh, Rup Lal

**Affiliations:** ^1^​ Post-Graduate Department of Zoology, Magadh University, Bodh Gaya, Bihar 824234, India; ^2^​ Department of Zoology, Deshbandhu College, University of Delhi, New Delhi, Delhi 110019, India; ^3^​ Department of Biotechnology, Jaypee Institute of Information and Technology, Uttar Pradesh, Noida 201309, India; ^4^​ Department of Zoology, Kirori Mal College, University of Delhi, Delhi 110007, India; ^5^​ Biotechnology and Viral Hepatitis Division, National Centre for Disease Control, New Delhi 110054, India; ^6^​ Deen Dayal Upadhyaya College, University of Delhi, New Delhi 110078, India; ^7^​ Shivaji College, University of Delhi, New Delhi 110027, India; ^8^​ Institute of Eminence, Delhi School of Public Health, University of Delhi, Delhi 110007, India; ^9^​ Acharya Narendra Dev College, University of Delhi, Govindpuri, Kalkaji, New Delhi 110019, India; ^10^​ Phixgen Pvt. Ltd., Sector 55, Noida, Uttar Pradesh, India

**Keywords:** host–protein interaction, MPXV, mutations, orthopoxvirus, phylogeny

## Abstract

While the world is still recovering from the Covid-19 pandemic, monkeypox virus (MPXV) awaits to cause another global outbreak as a challenge to all of mankind. However, the Covid-19 pandemic has taught us a lesson to speed up the pace of viral genomic research for the implementation of preventive and treatment strategies. One of the important aspects of MPXV that needs immediate insight is its evolutionary lineage based on genomic studies. Utilizing high-quality isolates from the GISAID (Global Initiative on Sharing All Influenza Data) database, primarily sourced from Europe and North America, we employed a SNP-based whole-genome phylogeny method and identified four major clusters among 628 MPXV isolates. Our findings indicate a distinct evolutionary lineage for the first MPXV isolate, and a complex epidemiology and evolution of MPXV strains across various countries. Further analysis of the host–pathogen interaction network revealed key viral proteins, such as E3, SPI-2, K7 and CrmB, that play a significant role in regulating the network and inhibiting the host’s cellular innate immune system. Our structural analysis of proteins E3 and CrmB revealed potential disruption of stability due to certain mutations. While this study identified a large number of mutations within the new outbreak clade, it also reflected that we need to move fast with the genomic analysis of newly detected strains from around the world to develop better prevention and treatment methods.

## Data Summary

**Fig. 1.**
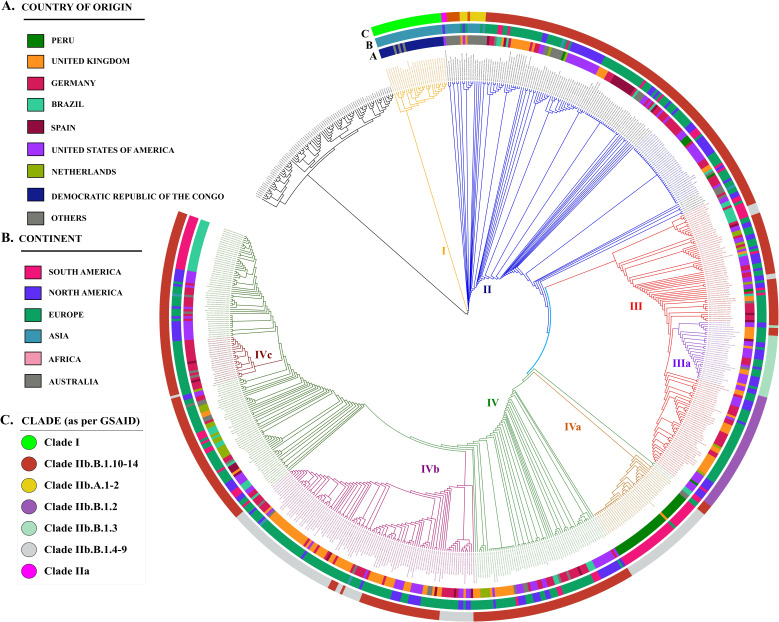
Whole-genome-based SNP phylogenetic tree representing the relationships among *Orthopoxvirus* isolates and 628 strains of MPXV. The tree is adorned with three concentric circles of colour-encoded information, Progressing outward from the centre: coloured circle A denotes the country of origin, coloured circle B indicates the continent of origin and coloured circle C displays clades as determined by the GISAID database.

The monkeypox isolates included in this study were downloaded from the GISAID (Global Initiative on Sharing All Influenza Data) database (https://gisaid.org/) on November 12, 2022. The GISAID accession ID numbers have been provided in the figures and in Table S1 (available with the online version of this article). The other members of genus *Orthopoxvirus* were downloaded from the National Center for Biotechnology Information (NCBI) database and their accession numbers are provided in Fig. 1.

**Impact Statement**

The emergence of monkeypox virus (MPXV) as a potential global outbreak poses a significant challenge to humankind, particularly as the world continues to recover from the Covid-19 pandemic. However, the lessons learned from the Covid-19 pandemic have emphasized the need for accelerated viral genomic research to facilitate the development of effective preventive and treatment strategies. In this context, this study focused on the evolutionary lineage of MPXV using whole-genome-based SNP and core-genome-based phylogenetic methods involving high-quality isolates primarily contributed from Europe and North America. Our findings revealed a substantial number of mutations in the core genome of newly emerged isolates. Along with this, we have also analysed the host–pathogen interaction network and identified key viral proteins, such as E3, SPI-2, K7 and CrmB, which play a crucial role in regulating the network and inhibiting the host's cellular innate immune system. Overall, our study highlights the urgent need to expedite genomic analysis of recently detected strains from diverse geographical regions, in order to enhance the development of improved preventive and therapeutic measures.

## Introduction

The discovery of the first monkeypox virus (MPXV) dates back to 1959 in cynomolgus monkeys during a transit from Singapore to Denmark [1]. However, the first reported incidence of monkeypox in humans occurred in 1970 in Basankusu Territory, Democratic Republic of Congo, Central Africa [1]. MPXV is a dsDNA virus belonging to the family *Poxviridae*, subfamily *Chordopoxvirinae* and the genus *Orthopoxvirus*, and is a close relative of vaccinia and cowpox viruses.

The human monkeypox is a viral zoonotic disease that exhibits self-limiting characteristics and predominantly affects individuals residing in close proximity to tropical rainforests and who engage in the consumption of bushmeat [2, 3]. The primary routes of transmission are through direct contact with the blood, bodily fluids, and lesions of infected animals, as well as via shedding of viral particles in feces and contamination of shared items.

The genus *Orthopoxvirus* includes a variety of viruses, such as the vaccinia virus, cowpox virus, camelpox virus, rabbitpox virus, horsepox virus, ectromelia virus, variola virus, buffalopox virus and Akhmeta virus, among others. The evolutionary study of genomes of *Orthopoxvirus* has been a topic of extensive research in recent years. A recent study on five newly isolated cowpox virus genomes from cats and humans, in combination with other members of the family *Orthopoxvirus*, revealed that cowpox virus is not a monophyletic entity, but rather a polyphyletic assemblage comprising several distinct species [4]. Another notable study of the evolutionary dynamics of *Orthopoxvirus* genomes was the origin and evolutionary trends of accessory genes in the genus *Orthopoxvirus* [5]. The study found that most of the accessory genes were captured in early stages of *Chordopoxvirus* evolution, prior to the divergence of *Orthopoxvirus* and the sister genus *Centapoxvirus*. These genes were then subjected to extensive duplication and some were also gained during the evolution of *Orthopoxvirus* itself. However, nearly every accessory gene was lost in various *Orthopoxvirus* lineages, and no *Orthopoxvirus* retained them all.

Geographically, monkeypox has been endemic to central and western Africa, but in recent years, reports of human–human and nosocomial transmission have emerged, making MPXV a potential global threat, particularly in the wake of the Covid-19 pandemic [6, 7]. In response, the World Health Organization declared monkeypox a Public Health Emergency of International Concern on July 23 2022. Recently, this pathogen has resurfaced and as of January 23 2023, according to the reports from Centers for Disease Control and Prevention (USA), 114 987 cases of monkeypox have been confirmed in different parts of the world, including the USA (30 026 cases), Brazil (10 680 cases), Spain (7514 cases), France (4114 cases), Colombia (4062 cases), the UK (3735 cases), Peru (3711 cases), Mexico (3696 cases), etc. (https://www.cdc.gov/poxvirus/monkeypox/response/2022/world-map.html). The sudden emergence of MPXV and its widespread prevalence in more than 111 countries indicate that the virus must have been prevalent and been circulating at levels that have gone undetected by surveillance systems.

In order to tackle the outbreak of monkeypox, we need to drive focus on several fronts [8, 9]. The contribution of genomic analysis of SARS-CoV-2 (severe acute respiratory syndrome coronavirus 2) strains isolated from various geographical regions in combating the pandemic was of paramount importance. Not only did it serve as a strong aid to scientists but also it directed policy makers to combat the challenge in the best possible way [8, 9]. Undoubtedly, pathogen genomic studies have been very helpful in characterizing different circulating strains, identifying evolutionary links and predicting transmission patterns. In the present study, we thus attempted to analyse the phylogenetic position of the newly emerged and sequenced MPXV variants within the genus *Orthopoxvirus*. We particularly focused on the genomic variations present at protein level within MPXV strains, along with the generation of an interactome between human and MPXV. In addition, the amino acid level mutations in major regulatory hub proteins were analysed to determine their effect on the structural integrity of the virus. In conclusion, our comprehensive genomic analysis of newly emerged MPXV variants aimed at characterizing protein-level variations and evaluating the impact of regulatory hub protein mutations on the structural integrity of the virus, providing crucial insights into the evolutionary relationships and transmission patterns of this pathogen.

## Methods

### Selection of genomes

As of November 12 2022, a total of 3802 monkeypox viral genomes were present in the publicly available GISAID (Global Initiative on Sharing All Influenza Data) database (https://gisaid.org/), including the first monkeypox isolate (MpxV/cynomolgus monkey/USA/un-WRAIR7-61-P2/1962). However, the complete dataset of genomes consisted of 2906 individual genomes. These genomes were analysed and those with multiple instances of the nucleotide ‘N’ in their sequences were removed. Following retrieval of the sequences, quality assessment was performed using quast 5.0.2 [10], and out of the 2906 genomes available, 628 high-quality genomes were selected for downstream analyses (Table S1). In order to establish the phylogenetic position, using the whole-genome SNP method, all available genomes of genus *Orthopoxvirus* other than MPXV were downloaded (*N*=100). These comprised the sequences from cowpox viruses (*N*=41), vaccinia viruses (*N*=30), camelpox viruses (*N*=9), Akhmeta viruses (*N*=6), variola viruses (*N*=5) and ectromelia viruses (*N*=3). After the quality check, out of these 100 genomes, 85 were used for SNP-based phylogeny. Finally, the phylogenetic analysis was done using 713 viruses, 628 genomes of MPXV and the remaining 85 genomes of other viruses belonging to the genus *Orthopoxvirus*.

### Genome annotation

The genomes of 628 MPXVs were annotated using Prokka rapid prokaryotic genome annotation [11]. To further refine the database, the prokka-genbank_to_fasta_db tool was used to generate a MPXV database. The GenBank full format files of isolates, namely Congo_2003_358, DRC_06–1070, MPXV-WRAIR7-61, COP-58, Israel_2018, Sudan_2005_01, Cote_d'Ivoire_1971, Liberia_1970_184 and Zaire-96-I-16, were downloaded from the National Center for Biotechnology Information (NCBI) and the database was formatted. This database was further used to annotate the MPXV genomes in Prokka using --kingdom Viruses --gcode 1 flags.

### Phylogeny

The whole-genome SNP-based phylogenetic tree was reconstructed using kSNP4 [12]. The optimum *k*-mer size (*k*-mer=19) of the dataset was determined using kchooser, a program that measures the diversity of sequences in the dataset. The whole-genome consensus parsimony tree (-min_frac 0.2) was reconstructed in kSNP4 and visualized in iTOL [13]. Additionally, to access the similarity based on core genome, the multifasta core-genome alignment was generated using prank [14] in Roary [15] with minimum percentage identity of 95 %. The alignment file was used to reconstruct the phylogenetic tree using the maximum-likelihood statistical method in iq-tree2 [16].

### Analysis of the evolutionary dynamics of core genes

The GenBank files generated using Prokka were subjected to the get_homologues package for analysis of the core genome using the OrthoMCL clustering algorithms with minimum identity and query coverage of 95 % [17]. Furthermore, the substitution rates at non-synonymous and synonymous sites were determined for these core genomes. Using muscle v3.8.31 and HyPhy v2.2.4 [18], orthologous gene clusters were aligned and stop codons were removed. For each orthologous gene cluster, Datamonkey v2.0 [19] (http://www.datamonkey.org/slac) used the single-likelihood ancestor counting (SLAC) method to calculate the dN/dS value. The dN/dS values were plotted using ggplot2 in R (R Development Core Team, 2015). We validated the dN/dS analysis utilizing the potion package version 1.1.3 [20], which employs the Codeml program to detect indications of positive selection through site-model analysis. To achieve this, the orthologous gene clusters were determined using OrthoMCL version 1.4 [21], and the orthologous groups were then screened for positive selection. Recombination was detected and removed from analysis using PhiPack [22], which incorporates three recombination tests: Phi, NSS and Max Chi2. Multiple protein sequence alignments were generated for each group using muscle version 3.8.31, followed by trimming using TrimAl version 1.2 [23]. The subsequent methodology was similar to our previous study [24]. Furthermore, we clustered 628 monkeypox virus proteins at 95 % sequence similarity and query coverage using cd-hit [25], and the resultant pangenome protein sequences were used for host–pathogen protein–protein interaction (PPI) analysis.

### Modelling of the host–pathogen interaction (HPI) network

Viruses rely on interactions between host and viral proteins to carry out all their life cycle functions, including infection, replication and even the assembly of new viral particles [9]. MPXV protein sequences were submitted to HPIDB3.0 [26], the Host–Pathogen Interaction Database, to predict their direct interaction with humans as the principal host. blastp was used to retrieve homologous host/pathogen protein sequences [27]. For high-throughput analysis, blastp was used to search multiple protein sequences at once, and the results were presented both in a tabular format and as sequence alignments [26, 27]. Cytoscape v3.9.1 was used to construct and visualize the HPI network [28]. The constructed network demonstrated proteins with the highest degrees that interact with a large number of signalling proteins played a key regulatory role as hubs. The hub proteins were identified using Network Analyzer [29], a plugin of Cytoscape v3.9.1.

### Computational structure analysis of key monkeypox viral proteins

Based on the host–pathogen analysis, a few key proteins like protein E3, cytokine response-modifying protein B (CrmB), SPI-2, protein K7 and protein E8 seemed to be of interest. Computational structural analysis was carried out to understand the effect of mutation on the key viral proteins E3 and CrmB. The computational structures of wild-type and mutant proteins E3 and CrmB were constructed using the Phypre2 [30] and Swiss model [31]. The structures were energy minimized by the Chiron energy minimization server [32]. The structures were also validated using the Ramachandran plot. The effects of the mutations were analysed using FoldX [33]. The structures were repaired before building the mutant models. Repair protein structure helped identify residues with poor torsion angles or Van der Waal’s classes or total energy, and repaired them. The FoldX tool provided the difference in Gibbs energy of the protein.

## Results and Discussion

### General genomic attributes

MPXV belongs to genus *Orthopoxvirus* with a mean genome size and coding sequences of 197.24±0.695 kb and 211.097±1.16, respectively, and the mean mol% G+C was 33.00±0.028 mol% (Table S1). The results are in consensus with previously published reports [34]. The majority of high-quality MPXV isolates included in this study are from Germany (*n*=141), the USA (*n*=137), the UK (*n*=120), Brazil (*n*=56), Peru (*n*=42), Spain (*n*=28), the Netherlands (*n*=27) and the Democratic Republic of the Congo (*n*=24) (Table S1).

The distribution of these isolates by continents revealed that out of 628, 342 isolates were from Europe, 139 from North America, 99 from South America, 36 from Africa, 11 from Asia and only 1 from Australia (Table S1). Out of all the isolates, 590 were isolated in the year 2022. In fact, all the isolates from Europe were isolated in 2022 from the current outbreak. These datasets suggest that the likelihood of virus isolation is higher in Europe and North America, which may be attributed to several factors. One possibility is the increased transmission rate of the virus in these regions, resulting in more cases and therefore more isolates. Additionally, the existence of efficient tracking systems in Europe and North America may have contributed to the high number of isolates collected from these regions.

However, it is interesting to note that despite a high population density, Asia reported a lower number of high-quality genomes in comparison to other regions. This may be due to a multitude of factors, including variations in testing practices or tracking systems, which may have led to fewer cases being identified and, subsequently, fewer isolates being collected.

All the isolates included in this study are of human origin, except the reference strain (MpxV/cynomolgus monkey/USA/un-WRAIR7-61-P2/1962), which was isolated from *Macaca fascicularis* in the year 1962. In all likelihood, the first disease transmission took place via exotic animals transported from tropical rainforests to other parts of the western hemisphere [35].

### Phylogenetic analysis

A phylogenetic tree based on whole-genome SNPs representing 16 different viruses was reconstructed for the members of the genus *Orthopoxvirus*, including ectromelia (*n*=3), abatino (*n*=1), akhmeta (*n*=4), cetacean (*n*=1), raccoonpox (*n*=1), skunkpox (*n*=1), volepox (*n*=1), cowpox (*n*=35), variola (*n*=4), tetrapox (*n*=1), camelopox (*n*=9), vaccinia (*n*=15), horsepox (*n*=2), rabbitpox (*n*=1), buffalopox (*n*=6) and monkeypox (*n*=628) viruses. MPXV clustered distinctly from other members of the genus *Orthopoxvirus* but closely with the camelopox virus (Fig. 1). The camelopox virus clade comprised other viruses namely tetrapox virus, variola virus and cowpox virus.

The 628 strains of MPXV included in this study represent multiple clades and subclades as assigned by the GISAID phylogeny (Fig. 1). The clades and subclades designated in GISAID phylogeny are as follows: I (probable IIb A) (*n*=27), IIa (probable IIb A) (*n*=2), IIb A (*n*=6), IIb A.1 (*n*=4), IIb A.2.1 (*n*=3), IIb A.2.2 (*n*=1), IIb A.2.3 (*n*=1), IIb B.1 (*n*=292), IIb B.1.1 (*n*=72), IIb B.1.2 (*n*=47), IIb B.1.3 (*n*=25), IIb B.1.4 (*n*=2), IIb B.1.5 (*n*=7), IIb B.1.6 (*n*=36), IIb B.1.7 (*n*=47), IIb B.1.8 (*n*=13), IIb B.1.9 (*n*=4), IIb B.1.10 (*n*=7), IIb B.1.11 (*n*=15), IIb B.1.12 (*n*=5), IIb B.1.13 (*n*=5) and IIb B.1.14 (*n*=7), where *n* is the number of isolates used in this study representing each clade (Fig. 1). Clade IIb B.1 is the most prevalent with 292 isolates, with the majority of isolates from Europe (*n*=193), followed by North America (*n*=61), South America (*n*=33), Asia (*n*=4) and Australia (*n*=1). Other notable clades include clade IIb B.1.1, with 72 isolates, primarily found in Europe (*n*=33) and South America (*n*=22), and clades IIb B.1.2 and IIb B.1.7, each with 47 isolates. The remaining clades have varying number of isolates, ranging from 36 to 1.

In particular, a total of four major clusters have been identified when delineating the 628 MPXV isolates using the SNP-based whole-genome phylogeny method. As for the GISAID, the clade I in our study also represents 27 isolates and the isolate hMpxV/DRC/USAMRIID-07-0104/2006 (EPI_ISL_13056243) clustered to the camelopox virus clade, which includes other viruses such as tetrapox virus, variola virus and cowpox virus. Interestingly, the first MPXV isolate (MpxV/cynomolgus monkey/USA/un-WRAIR7-61-P2/1962) failed to cluster with other isolates in clade I. Instead, it clustered in a separate clade (clade IIa as per GISAID) along with an isolate from Liberia (hMpxV/Liberia/CDC-184/1970), which was isolated in 1970. This clearly suggests that the first isolate may have a distinct evolutionary lineage compared to the other isolates in clade I. It is also possible that the first isolate represents a different subtype of MPXV that has diverged from the other isolates over time.

A total of 141 MPXV isolates were identified as belonging to clade II, with representation from 18 countries. The distribution of these isolates among countries was as follows: the USA (*n*=35), Germany (*n*=33), the UK (*n*=18), Spain (*n*=13), Nigeria (*n*=7), Brazil (*n*=6), Austria (*n*=5), Peru (*n*=5), Belgium (*n*=4), with 2 isolates each from the Netherlands, Scotland and India, and 1 isolate each from Egypt, Liberia, Singapore, Israel, Japan, Mexico, France, Italy and the Philippines. This diversity in geographical distribution of clade II isolates may suggest a complex epidemiology and evolution of MPXV strains in different countries. As previously mentioned, the only non-human isolate (MpxV/cynomolgus monkey/USA/un-WRAIR7-61-P2/1962) included in the study clustered alongside the human isolate (hMpxV/Liberia/CDC-184/1970) in clade II.

The clade III of the MPXV isolates included in this study is represented by 131 strains, with the majority being from the USA (*n*=43), Germany (*n*=33), the UK (*n*=29) and Brazil (*n*=10). However, there does not appear a clear pattern of distribution of these strains based on their country of isolation, indicating a mosaic pattern of distribution and the presence of similar strains in different countries. A subclade, designated as IIIa, was identified within clade III and was found to be particularly prominent (Fig. 1). This subclade comprises a total of 29 isolates, with representation from the USA (*n*=15), the UK (*n*=9), Germany (*n*=3) and 1 isolate each from Spain and France. This clustering pattern and the geographical distribution of the isolates within subclade IIIa suggest the possible transcontinental transmission of similar strains, likely facilitated by patterns of travel.

In this phylogenetic study, a major clade designated as clade IV was identified, comprising 329 isolates. Further analysis of this clade revealed the presence of three distinct subclades, designated as IVa (*n*=38), IVb (*n*=102) and IVc (*n*=21). The distribution of isolates within clade IV is primarily dominated by strains from Germany (*n*=75), the UK (*n*=70), the USA (*n*=59), Brazil (*n*=40), the Netherlands (*n*=18) and Spain (*n*=11). Subclade IVa, which is represented by 38 isolates, displays a distinct geographical distribution pattern. The majority of isolates within this subclade are from Peru (*n*=35), with 2 isolates from Austria and 1 from the UK. This pattern of clustering suggests that there may be a specific genetic lineage or strain of the MPXV circulating in Peru, which has also been identified in Austria and the UK. This highlights the possibility of a recent transmission of this strain across geographical boundaries.

Similarly, subclade IVb, which comprises 102 isolates, was primarily dominated by strains from the UK (*n*=55), followed by Germany (*n*=22), the USA (*n*=16), Spain (*n*=4) and 1 isolate each from Mexico, Taiwan and Hong Kong. This geographical distribution of isolates within subclade IVb suggests the likelihood of a single isolate from the UK giving rise to distinct genetic lineages that have been transmitted across continental boundaries; specifically, from Europe to North America and Asia.

Subclade IVc, representing 21 isolates, was a minor subclade within clade IV. Unlike subclades IVa and IVb, this subclade is primarily represented by European isolates, with the majority originating from Germany (*n*=17) and 2 from Spain, with an additional 1 isolate each from Scotland and Austria. This indicates that the genetic lineage or strain represented by subclade IVc has a predominantly European distribution and may have evolved independently. It is worth noting that the small number of isolates in this subclade may not be representative of the true distribution of this strain in Europe and demands further studies with a still larger sample size.

**Fig. 2.**
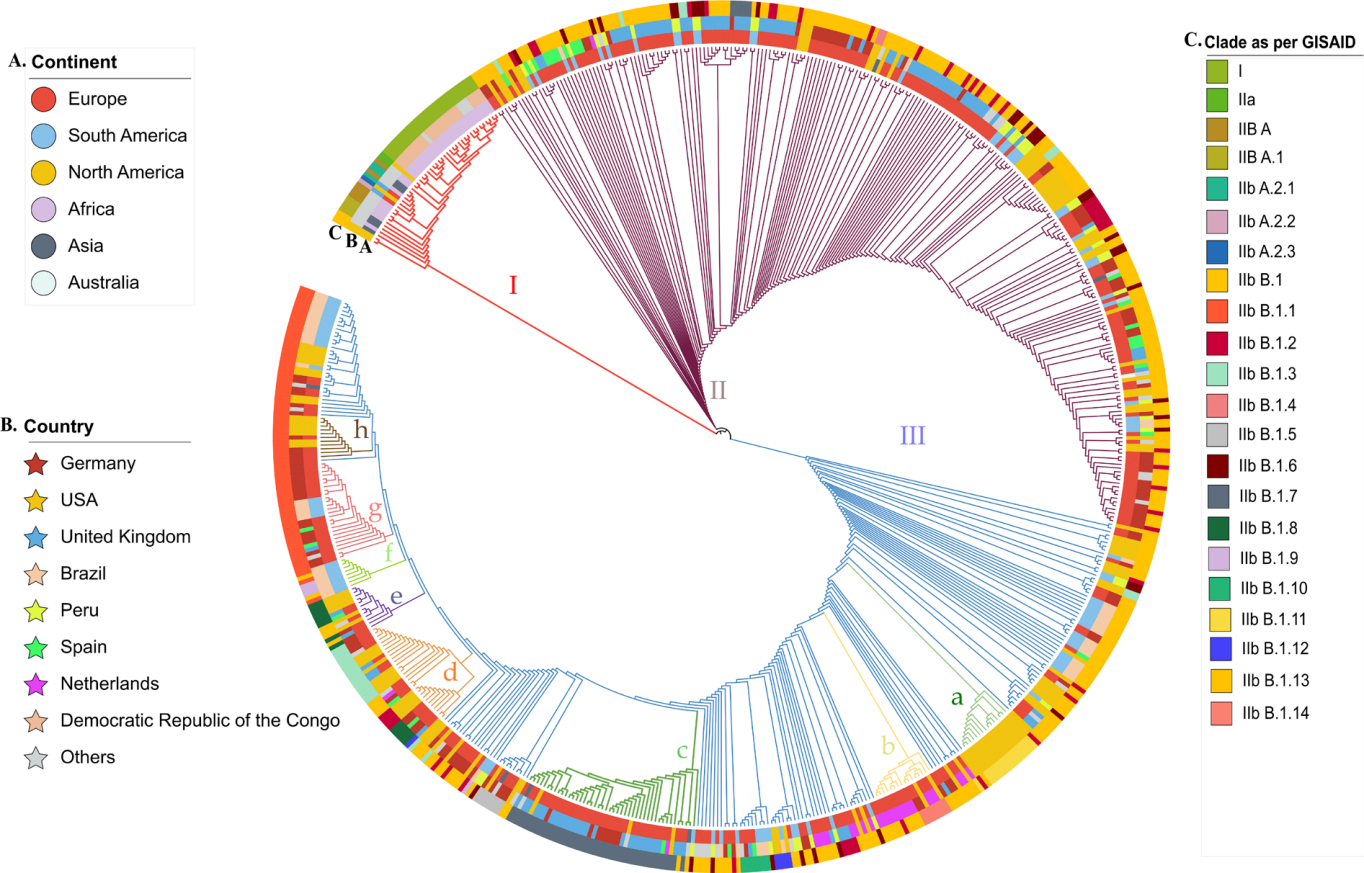
Core-genome-based phylogenetic tree depicting the relationships among 628 strains of MPXV. The tree is decorated with three concentric circles of colour-coded Information, moving outward from the centre: coloured circle A specifies the country of origin, coloured circle B indicates the continent of origin and coloured circle C shows clades as determined by the GISAID database.

In this study, we employed another phylogenetic tree reconstruction method using the core-genome sequences (Fig. 2). Our analysis revealed the presence of three major clades, designated as clade I, clade II and clade III. Clade III was further subdivided into several subclades, labelled IIIa through IIIh in Fig. 2. Clade I comprised 47 isolates originating from Africa (*n*=36), Asia (*n*=5), North America (*n*=4) and Europe (*n*=2). Notably, the first identified MPXV isolate (MpxV/cynomolgus monkey/USA/un-WRAIR7-61-P2/1962) clustered within clade I, in close proximity to an isolate from Liberia, which is concordant with the results obtained from SNP-based phylogeny. Upon comparison of the classification of the GISAID clade and the SNP-based phylogeny, the core-genome phylogeny clustered together the members from clades I (*n*=27), IIa (*n*=2), IIb A (*n*=6), IIb A.2.1 (*n*=3), IIb A.2.3 (*n*=1), IIb A.2.2 (*n*=1), IIb A.1 (*n*=4) and IIb B.1 (*n*=3). This suggests that the core genome of MPXV possesses a high degree of conservation within these subclades, and that the genetic variation observed in the SNP-based phylogeny may be due to other regions of the genome. This is an important finding as it highlights the importance of using multiple methods to decipher the evolutionary relationships of a pathogen like MPXV, and can be a useful information for tracking the spread of the virus and understanding its transmission dynamics.

Likewise, clade II exhibited 245 isolates, primarily sourced from Europe (*n*=166), North America (*n*=42) and South America (*n*=34). The majority of these isolates belonged to the IIb B.1 clade of GISAID (*n*=167), with additional representation from IIb B subclades such as IIb B.1.2 (*n*=27), IIb B.1.6 (*n*=25), IIB B.1.3 (*n*=7), etc. The countries with the highest representation within this clade were the UK (*n*=74), Germany (*n*=55), the USA (*n*=42), Peru (*n*=29) and Spain (*n*=18), indicating a similarity in lineage distribution across geographical regions.

The clade III classification comprises a total of 336 isolates, which are geographically distributed across Europe (*n*=191), North America (*n*=92), South America (*n*=51) and Asia (*n*=2). The countries with the highest number of contributing isolates were the USA (*n*=90), Germany (*n*=65), the UK (*n*=43), Brazil (*n*=42), Ireland (*n*=38) and the Netherlands (*n*=28). An analysis of the GISAID clade classification in the core tree revealed the following distribution of subclades within clade III: IIb B.1 (*n*=148), IIb B.1.1 (*n*=60), IIb B.1.7 (*n*=40), IIb B.1.3 (*n*=20), IIb B.1.2 (*n*=16), IIb B.1.11 (*n*=16), IIb B.1.8 (*n*=9), IIb B.1.6 (*n*=8), etc.

The present study also identified several subclades within clade III of MPXV using core-genome phylogeny. A detailed analysis of the subclades revealed that subclade ‘a’ was found to be exclusively composed of 15 isolates from the USA. Subclade ‘b’, however, had a total of 16 isolates, with the majority (*n*=11) being identified from the Netherlands. The largest subclade (subclade ‘c’) was found to comprise 45 isolates, with the majority of them being from the UK (*n*=29). Germany also contributed a significant number of isolates (*n*=8) to this subclade. Subclade ‘d’ was composed of 30 isolates, with the majority of them being from the USA (*n*=13). This subclade also included isolates from Germany (*n*=8), the UK (*n*=4), Spain (*n*=2), the Netherlands (*n*=1), Austria (*n*=1), and France (*n*=1).

Similarly, subclades ‘e’ and ‘f’ were composed of 12 and 8 isolates, respectively. Notably, all the isolates in subclade ‘f’ were from Brazil. Subclade ‘g’ was found to comprise 26 isolates, with the majority of them being from Germany (*n*=17) and Brazil (*n*=5). Lastly, subclade ‘h’ was found to comprise 11 isolates, with the majority of them being from the USA (*n*=7) and 4 from Germany.

Overall, these results suggest that the MPXV has a diverse geographical distribution, with different subclades showing a distinct lineage that is either confined to a particular region or has already become globally distributed. The dominance of a particular subclade in a specific population can have important implications for the development of appropriate treatment methods. Therefore, it is essential to continue monitoring the genetic diversity of MPXV in order to better understand the epidemiology and evolution of this virus.

### Evolutionary dynamics of MPXV

**Fig. 3.**
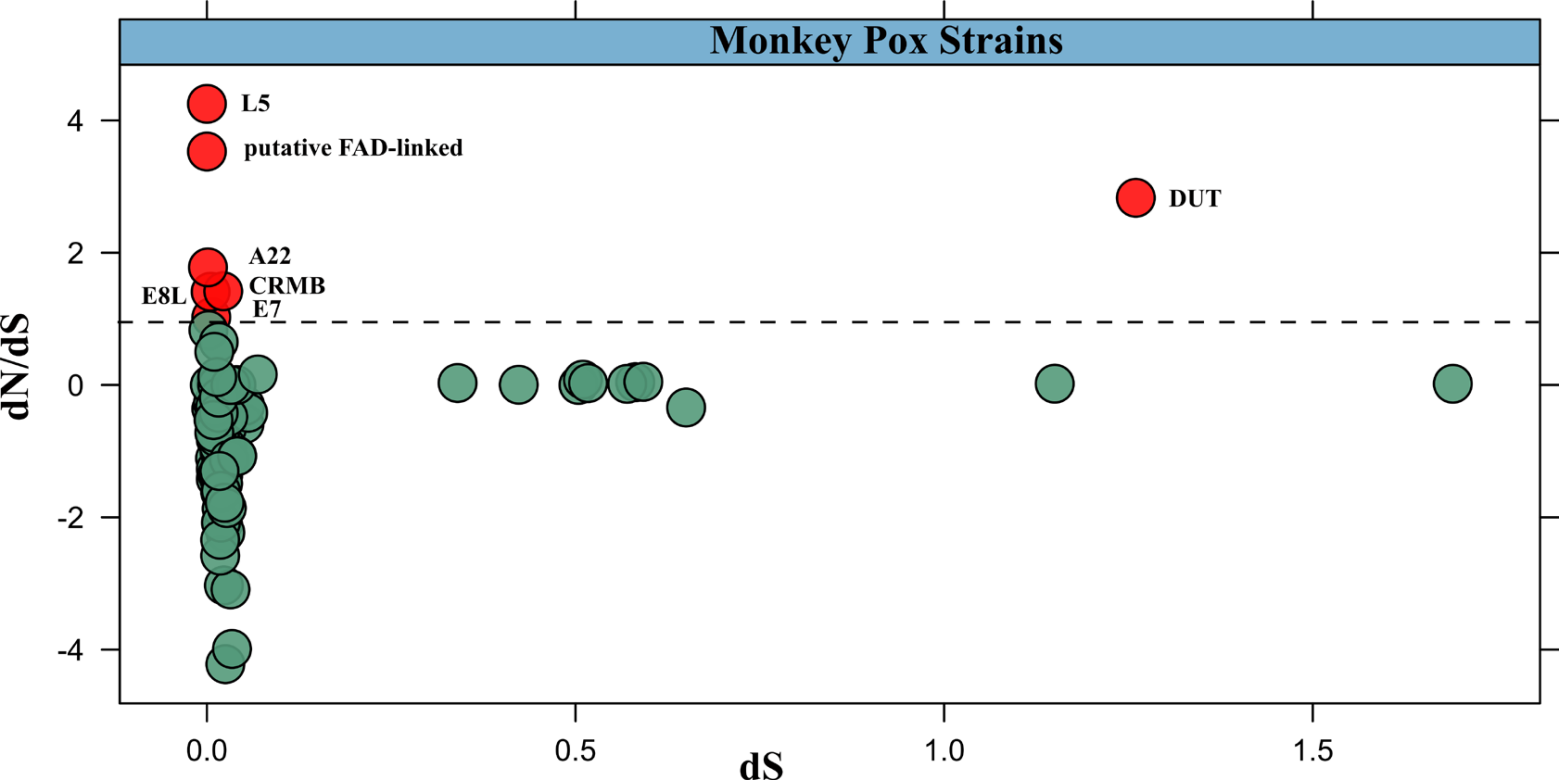
The dN/dS analysis of core genes for estimating the direction of selection.

Next, we analysed the core genome of MPXV. A total of 118 genes were conserved among the MPXV isolates (Fig. 3, Table S2). Among the 118 conserved genes, 41 encode hypothetical proteins; all genes were further confirmed not to be pseudogenes using blastp with COG functions (Table S2). Furthermore, we analysed the mutations in the core genome and we found that 93 genes out of 118 harboured different types of mutations such as point mutations, frameshift mutations and deletion mutations (Table S3). We then specifically used these core genes to quantify selection pressure. The dN/dS metric is one of the most widely used methods of quantifying selection pressure, and compares synonymous and non-synonymous substitutions. This method is frequently used to determine whether a protein is subject to purifying selection (dN/dS <1), is evolving neutrally (dN/dS ≈ 1) or is undergoing positive, diversifying selection (dN/dS >1) [36]. In the MPXV core genome, we identified seven proteins that were showing diversifying selection. These proteins included L5, A22, DUT, E8L, E7, CrmB and putative FAD-linked protein (Fig. 3, Tables S3 and S4). The L5 protein and E8L (dN/dS ≈ 1.41) are late viral, outer membrane proteins, which mediate attachment specifically to glycosaminoglycans and play an important role in membrane fusion with the host cell [34]. In the later stages of infection, the L5 protein could serve as a potential drug target predicted based on heterology to human proteins, solubility and antigenicity [35, 36]. L5 (dN/dS ≈ 4.25) is still moving towards achieving the optimal state of mimicry, which could help the virus to evade the innate immune response. The result agrees with a previous study by Swetha *et al.* (2022), which also reported the binding ability of L5 protein with MHC class-II molecules; we suggest the evolution of L5 protein epitopes might be changed in response to the direct drug induction of the host population [37]. Additionally, the heterodimer constituent of DNA polymerase, i.e. protein A22, is positively selected and it has been reported to be involved in the processivity of DNA replication with E4 protein and DNA substrates [38]. Furthermore, it has been demonstrated that due to the absence of proliferating cell nuclear antigen (PCNA; required for processivity), the A22–E4 heterodimer forms a complex holoenzyme in MPXV responsible for high processivity of DNA synthesis [39], and its greater purifying selection (dN/dS ≈ 1.77) indicates that the gene is evolving in MPXV during evolutionary processes in order to enhance its multiplicity. Likewise, the CrmB protein (dN/dS ≈ 1.12) is also under purifying selection, which may provide a selective advantage to the MPXV to protect the infected cells from the host antiviral response. A cytokine secreted by T cells and macrophages, tumour necrosis factor alpha (TNF-α), protects cells from viral infection and can kill infected cells [40]. The CrmB protein binds to TNF-α and TNF-β and, thus, protects the infected cells by preventing a TNF-mediated immune response against viruses [40]. Furthermore, the two other positively selected proteins, i.e. the protein E7 (dN/dS ≈ 1.02) and the putative FAD-linked protein (dN/ dS ≈ 3.53), might be evolving to help the virus to overcome intracellular host restrictions and achieve efficient survival.

### Exploring the network for HPI between MPXV and its host

**Fig. 4.**
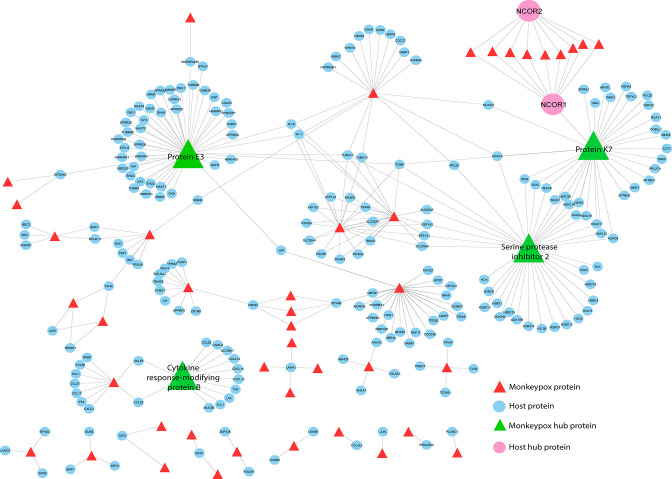
HPI network. MPXV protein sequences were submitted to the Host–Pathogen Interaction Database (HPIDB3.0), to predict direct interactions with humans as the principal host. The HPI network was constructed and visualized using Cytoscape v3.9.1. Hub proteins were identified using Network Analyzer, a plugin of Cytoscape v3.9.1.

A detailed study of the interplay between poxvirus proteins and the host immune system is of great interest to understanding the infectivity of these viruses, and will also pave the way for successful design and administration of recombinant vaccines. The HPI network of MPXV contained 331 edges and 273 nodes, including 55 viral and 218 host proteins (Fig. 4). The significant existence of a few hubs, namely, protein E3, serine protease inhibitor 2 (SPI-2), protein K7 and CrmB in the network, and the attraction of a large number of low-degree nodes toward each hub showed strong evidence of control of the topological properties of the network by a few hub proteins. Protein E3 was found to have a connection with 47 human host proteins, whereas SPI-2, protein K7 and CrmB exhibited 37, 33 and 21, respectively (Fig. 4). These monkeypox viral proteins were the main hubs in the network, which regulate/control the network. Based on degree distribution, the viral protein E3 showed the highest interaction, followed by serine protease inhibitor 2, protein K7 and CrmB.

Protein E3 plays a critical role in unhindered viral replication by blocking the cellular innate immune system [41]. Generation of IFNs is the prime response against viral infection. E3 protein of MPXV is known to produce IFN-resistant phenotypes by inhibiting the phosphorylation of PKR and eIF2α [42], which is in accordance with our interactome study where E3 is interacting with host EIF2AK2. Furthermore, the interactome analysis also showed that E3 protein interacts with host interleukin enhancing binding factors 2 and 3 (ILF2/3), which are known for providing an innate antiviral response by regulating the transcription of the IL-2 gene during T cell activation [43]. Furthermore, it is known that the suppression of cognate T cell activation, which evades CD8^+^ and CD4^+^ responses, is one of the immune escape mechanisms of MPXV [44].

Our study has shown that viral protein K7 interacts with several host proteins like NCOR1, SPIRE1, DDX3X, WNK1/2/3, SNX5, etc. K7 is known as an antagonist of innate immunity and acts as a virulence factor that inhibits IFN-regulatory factor 3 (IRF3) and NFκB activation [45]. Studies on the vaccinia virus have also shown that K7 protein interacts with SPIR-1, which is a virus restriction factor and activates innate immune signalling that is critical for the host response against viral infection [46]. Additionally, our interactome analysis revealed the interaction between K7 and DEAD-box protein 3 (DDX3). In the viral infection, the viruses are detected by several pattern recognition receptors (PRRs) like TLRs, RIG-like helicases, etc., which promotes antiviral activity by inducing IFN-β production through the activation of IRF3 and IRF7. Studies on vaccinia virus have shown that interaction between K7 protein and DDX3 inhibits PRR-induced IFN-β induction by suppressing TBK1/IKKɛ-mediated IRF activation [47].

K7 of vaccinia virus also interacts with the WNK (with-no-lysine) family and plays a crucial role in the antiviral immune response and knockdown of WNK family members, resulting in increased growth of vaccinia virus in the host. WNK 1 and WNK 3 stimulate IL-1 by activating p38 kinase, which in turn is inhibited by co-expression of K7 [48]. Interestingly, our interactome study between human and MPXV also showed an interaction between K7 and WNK family members. The PPIs also showed that two host proteins, NCOR1 and NCOR2, exhibit a maximum interaction with viral proteins. NCOR1 interacting with viral hub protein K7 showed an important co-relation with infections caused by MPXV. Both NCOR1 and NCOR2 are responsible for the repression of transcription by promoting histone deacetylation and chromatin repression, impeding access to transcription factors [49] and, hence, can be proposed to bring about gene silencing in the host upon a viral infection.

In the activation of antiviral immune response by the host, TNF plays a pivotal role, where it either acts directly as cell-death-inducing cytokine on virus-infected cells or indirectly as an inducer of the innate and adaptive immune response against the invading virus. Viruses have co-evolved with the host for their better survival and devised several strategies to evade TNF-mediated responses. One such strategy is the generation of cytokine response modifying protein B (CrmB), which is an excellent immunomodulator. Studies have shown that CrmB binds with TNF and several chemokines like CCL25, CCL28, CXCL12β, CXCL13 and CXCL14 to inhibit host immune responses against the virus [49, 50]. The binding of CrmB with chemokines prevents recruitment of T cells and B cells, dendritic cell migration to epidermal tissue, and recruitment of B cells to spleen and lymph nodes [51]. Interestingly, our PPI results also showed the interaction of CrmB protein of MPXV with host TNF and several chemokines like CCL1, CCL21, CCL25, CXCL13, CXCL14, etc. Another key protein hub, SPI-2, also contributes to poxvirus immune escape. By targeting caspase-1, SPI-2 prevents apoptosis and cytokine activation. Furthermore, the induction of IFN-β and its downstream genes is inhibited by the ectopic expression of SPI-2; thus, preventing the host conferring an IFN-mediated immune response against viruses.

### Computational analysis of the 3D structures of proteins E3 and CrmB

**Fig. 5.**
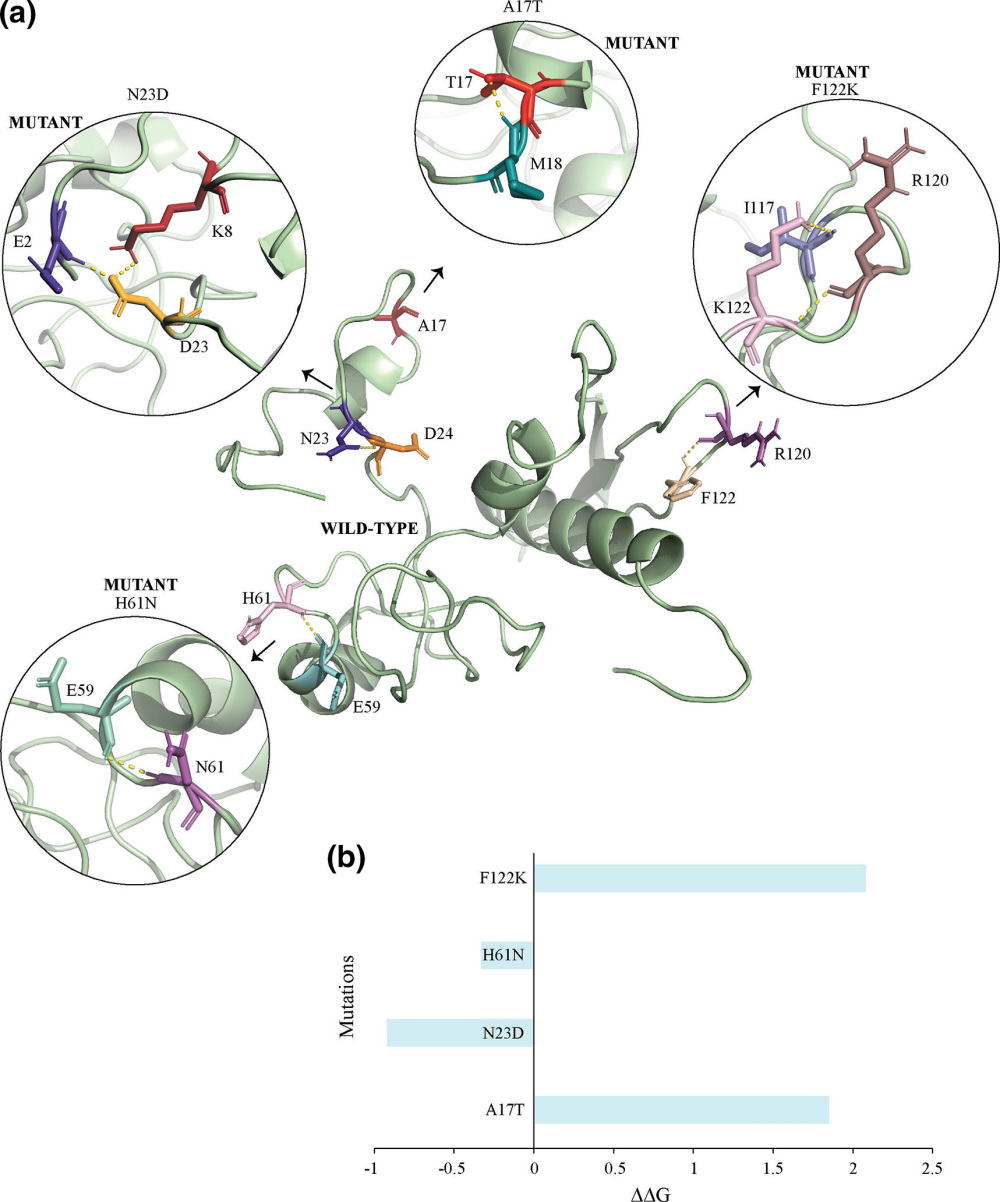
Structure analysis of protein E3. (**a**) Structures depicting mutations, specifically F121K, H61N, N23D and A17T. (**b**) ΔΔG analysis to illustrate the stability of mutations. Mutations with ΔΔG higher than zero will destabilize the structure, while mutations with ΔΔG lower than zero will stabilize it.

**Fig. 6.**
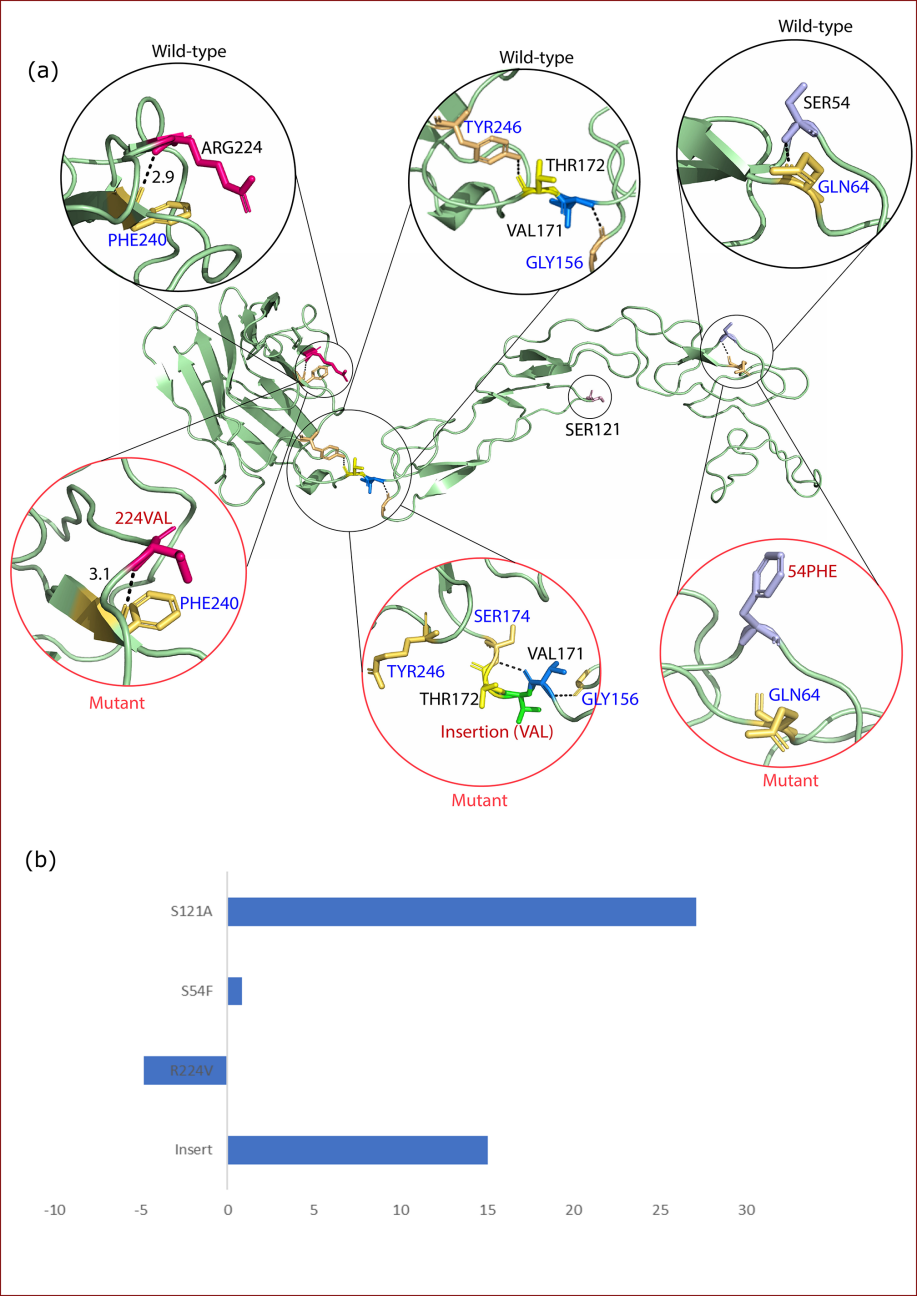
Structure analysis of CrmB protein. (**a**) Structures depicting mutations, specifically S121A, S54F, R224V and an insertion of valine (V) at position 172. (**b**) ΔΔG analysis to depict the stability of mutations.

Computational structural analysis was performed to investigate the impact of mutations on key viral proteins, specifically protein E3 and CrmB. Due to the lack of structurally similar templates for a significant portion of the proteins, Phyre2 was utilized to generate 3D structures of protein E3 and CrmB (Figs 5 and 6). In the E3 protein, hydrogen bond analysis using PyMOL revealed that mutations A17T, N23D and F122K resulted in a gain of hydrogen bonds (Fig. 5a). Protein stability is determined by free energy, with lower values indicating greater stability. The change in free energy (ΔΔG) between the wild-type and mutant forms was calculated, with mutations resulting in a ΔΔG greater than zero destabilizing the protein and those with a ΔΔG less than zero stabilizing it. Of the four mutations in protein E3 (A17T, N23D, H61N and F122K), A17T and F122K destabilized the protein, while N23D and H61N stabilized it (Fig. 5b). Protein E3 plays a crucial role in inhibiting the cellular innate immune system, allowing for unhindered viral replication [41]. IFNs are a critical component of the host innate immune response against viral infections. The E3 protein of MPXV has been shown to elicit an IFN-resistant phenotype through inhibition of phosphorylation of the PKR and eIF2α proteins, a mechanism of action that has been well documented in the literature [42].

Similarly, in the CrmB protein, the insertion of valine and S54F mutation resulted in a loss of hydrogen bonds, potentially disrupting the protein’s structure (Fig. 6a). Of the four mutations in CrmB (S121A, S54F, R224V and an insertion of valine at position 172), S121A, S54F and the insertion of valine destabilized the protein, while R224V stabilized it (Fig. 6b). CrmB protein has a smallpox virus-encoded chemokine receptor (SECRET) domain that dispenses chemokine inhibitory activities, and allows the virus to differentially block chemokines and TNFs [51]. CrmB host protein interacts with the host TNFs via its N-terminal domain [52]. It also interacts with calcium modulating ligand (CAMLG), which takes part in the calcium signal transduction pathway [51]. Furthermore, it interacts with mucin 5B protein, a glycosylated macromolecular component of mucus secretions. The protein K7, a part of the virulence and infection proteins, interacts with the DEAD-box RNA helicase DDX3, TNF-associated factor 6 and IL-1 receptor associated kinase to inhibit activity of IFN regulatory factors [45].

### Conclusion

In light of the recent surge in human monkeypox cases worldwide, it is crucial to investigate the mutational rate, virulence and evolutionary lineage of different isolates from various geographical locations. Utilizing the SNP-based whole-genome phylogeny method, four major clusters were identified among 628 MPXV isolates. These clusters exhibited diverse geographical distributions and unique evolutionary lineages, suggesting a complex epidemiology and evolution of MPXV strains. Furthermore, a study of the HPI network revealed key viral proteins, including E3, SPI-2, K7 and CrmB, that act as hubs in regulating the network. The 3D structural analysis of proteins E3 and CrmB revealed that certain mutations can potentially disrupt protein stability, providing new insights into the infectivity of poxviruses and potential targets for recombinant vaccine development.

## Supplementary Data

Supplementary material 1

Supplementary material 2
