# Supplementary material for: Monkeypox virus: phylogenomics, host–pathogen interactome and mutational cascade
Source: Microb Genom. 2023 Apr 12;9(4):mgen000987. doi: 10.1099/mgen.0.000987 (PMC10210936; doi:10.1099/mgen.0.000987)
Supplement: Supplementary material 1 [file mgen-9-987-s001.pdf]

Table S1: General Genomic Attributes of Monkeypox Virus.

S. No.	Virus Strain	GISAID Accession ID	Location	Host	Contigs	Total Length (bp)	GC Content (%)	Specimen	Clade Lineage as per GISAID
1	hMpxV/Germany/BY-IMB-25241/2022	EPI_ISL_13052263	Europe / Germany / Bavaria	Human	1	197378	32.99	Skin swab	IIb B.1
2	hMpxV/France/un-UT-67/2022	EPI_ISL_13052275	Europe / France	Human	1	197103	33.02	Not Available	IIb B.1.10
3	hMpxV/Belgium/UZ_Rega_1/2022	EPI_ISL_13052282	Europe / Belgium	Human	1	198010	32.93	Not Available	IIb B.1
4	hMpxV/Belgium/UZ_Rega_2/2022	EPI_ISL_13052283	Europe / Belgium	Human	1	198016	32.93	Not Available	IIb B.1
5	hMpxV/France/un-VGEMI-HCL0001/2022	EPI_ISL_13052287	Europe / France	Human	1	197120	33.01	Skin swab	IIb B.1.3
6	hMpxV/USA/MA-CDC-001/2022	EPI_ISL_13052289	North America / USA / Massachusetts	Human	1	197205	33.00	Not Available	IIb B.1
7	hMpxV/Australia/VIC-VIDRL01/2022	EPI_ISL_13052292	Oceania / Australia / Victoria	Human	1	197443	32.97	Not Available	IIb B.1
8	hMpxV/DRC/CDC-005/1978	EPI_ISL_13053218	Africa / Democratic Republic of the Congo	Human	1	196967	33.09	Not Available	I (probable IIb A)

9	hMpxV/DRC/USAMRIID-06-0950/2006	EPI_ISL_130 56233	Africa / Democratic Republic of the Congo	Human	1	196440	33.10	Scab or vesicle	I (probable IIb A)
10	hMpxV/DRC/USAMRIID-06-0970/2006	EPI_ISL_130 56234	Africa / Democratic Republic of the Congo	Human	1	196740	33.09	Scab or vesicle	I (probable IIb A)
11	hMpxV/DRC/USAMRIID-06-0999/2006	EPI_ISL_130 56235	Africa / Democratic Republic of the Congo	Human	1	198597	33.11	Scab or vesicle	I (probable IIb A)
12	hMpxV/DRC/USAMRIID-06-1075/2006	EPI_ISL_130 56237	Africa / Democratic Republic of the Congo	Human	1	198877	33.11	Scab or vesicle	I (probable IIb A)
13	hMpxV/DRC/USAMRIID-06-1076/2006	EPI_ISL_130 56238	Africa / Democratic Republic of the Congo	Human	1	198737	33.11	Scab or vesicle	I (probable IIb A)
14	hMpxV/DRC/USAMRIID-07-0045/2006	EPI_ISL_130 56239	Africa / Democratic Republic of the Congo	Human	1	197627	33.09	Scab or vesicle	I (probable IIb A)
15	hMpxV/DRC/USAMRIID-07-0046/2006	EPI_ISL_130 56240	Africa / Democratic	Human	1	197910	33.10	Scab or vesicle	I (probable IIb A)

			Republic of the Congo						
16	hMpxV/DRC/USAMRIID-07-0092/2006	EPI_ISL_13056241	Africa / Democratic Republic of the Congo	Human	1	197488	33.09	Scab or vesicle	I (probable IIb A)
17	hMpxV/DRC/USAMRIID-07-0093/2006	EPI_ISL_13056242	Africa / Democratic Republic of the Congo	Human	1	197632	33.09	Scab or vesicle	I (probable IIb A)
18	hMpxV/DRC/USAMRIID-07-0104/2006	EPI_ISL_13056243	Africa / Democratic Republic of the Congo	Human	1	197959	33.11	Scab or vesicle	I (probable IIb A)
19	hMpxV/DRC/USAMRIID-07-0120/2007	EPI_ISL_13056244	Africa / Democratic Republic of the Congo	Human	1	196740	33.09	Scab or vesicle	I (probable IIb A)
20	hMpxV/DRC/USAMRIID-07-0275/2007	EPI_ISL_13056245	Africa / Democratic Republic of the Congo	Human	1	196732	33.09	Scab or vesicle	I (probable IIb A)
21	hMpxV/DRC/USAMRIID-07-0283/2007	EPI_ISL_13056246	Africa / Democratic Republic of the Congo	Human	1	196730	33.09	Scab or vesicle	I (probable IIb A)

22	hMpxV/DRC/USAMRIID-07-0286/2007	EPI_ISL_13056247	Africa / Democratic Republic of the Congo	Human	1	197767	33.10	Scab or vesicle	I (probable IIb A)
23	hMpxV/DRC/USAMRIID-07-0287/2007	EPI_ISL_13056248	Africa / Democratic Republic of the Congo	Human	1	197346	33.09	Scab or vesicle	I (probable IIb A)
24	hMpxV/DRC/USAMRIID-07-0337/2007	EPI_ISL_13056249	Africa / Democratic Republic of the Congo	Human	1	196581	33.10	Scab or vesicle	I (probable IIb A)
25	hMpxV/DRC/USAMRIID-07-0354/2007	EPI_ISL_13056251	Africa / Democratic Republic of the Congo	Human	1	197147	33.11	Scab or vesicle	I (probable IIb A)
26	hMpxV/DRC/USAMRIID-07-0450/2007	EPI_ISL_13056252	Africa / Democratic Republic of the Congo	Human	1	196747	33.09	Scab or vesicle	I (probable IIb A)
27	hMpxV/DRC/USAMRIID-07-0480/2007	EPI_ISL_13056253	Africa / Democratic Republic of the Congo	Human	1	197347	33.09	Scab or vesicle	I (probable IIb A)
28	hMpxV/DRC/USAMRIID-07-0514/2007	EPI_ISL_13056254	Africa / Democratic	Human	1	197488	33.09	Scab or vesicle	I (probable IIb A)

			Republic of the Congo						
29	hMpxV/DRC/USAMRIID-07-0662/2007	EPI_ISL_13056255	Africa / Democratic Republic of the Congo	Human	1	196866	33.10	Scab or vesicle	I (probable IIb A)
30	hMpxV/Singapore/CDC-01/2019	EPI_ISL_13056274	Asia / Singapore	Human	1	197309	33.01	Not Available	IIb A.1
31	hMpxV/Nigeria/CDC-M5312_HM12/2018	EPI_ISL_13056282	Africa / Nigeria / Rivers State	Human	1	197209	33.03	Not Available	IIb A
32	hMpxV/Nigeria/CDC-M2957/2018	EPI_ISL_13056283	Africa / Nigeria / Lagos State	Human	1	197559	33.00	Not Available	IIb A
33	hMpxV/Nigeria/CDC-M2940_FCT/2018	EPI_ISL_13056284	Africa / Nigeria / Federal Capital Territory	Human	1	197547	33.00	Not Available	IIb A
34	hMpxV/Nigeria/CDC-M3021/2018	EPI_ISL_13056285	Africa / Nigeria / Delta State	Human	1	197556	33.00	Not Available	IIb A
35	hMpxV/Israel/IIBR-01/2018	EPI_ISL_13056289	Asia / Israel	Human	1	197417	33.00	Postule	IIb A.1

36	MpxV/cynomolgus monkey/USA/un-WRAIR7-61-P2/1962	EPI_ISL_13056556	North America / USA	Macaca fascicularis	1	199195	33.06	Not Available	IIa (probable IIb A)
37	hMpxV/Israel/IIBR-ISR001/2022	EPI_ISL_13056910	Asia / Israel	Human	1	196753	33.01	Not Available	IIb B.1
38	hMpxV/Republic of the Congo/CDC-358/2003	EPI_ISL_13058404	Africa / Republic of the Congo	Human	1	197195	33.09	Not Available	I (probable IIb A)
39	hMpxV/Liberia/CDC-184/1970	EPI_ISL_13058405	Africa / Liberia	Human	1	200256	33.09	Not Available	IIa (probable IIb A)
40	hMpxV/Sudan/UV-01/2005	EPI_ISL_13058459	Africa / Sudan / Nuri	Human	1	206372	32.95	Not Available	I (probable IIb A)
41	hMpxV/DRC/UV-001/1985	EPI_ISL_13058460	Africa / Democratic Republic of the Congo	Human	1	196487	33.08	Not Available	I (probable IIb A)
42	hMpxV/USA/TX-CDC-000/2021	EPI_ISL_13100621	North America / USA / Texas	Human	1	197265	33.01	Not Available	IIb A.2.3
43	hMpxV/France/un-IRBA-11/2022	EPI_ISL_13308158	Europe / France	Human	1	196172	33.01	Buccal swab	IIb B.1
44	hMpxV/Brazil/SP-IAL-03/2022	EPI_ISL_13314740	South America / Brazil / Sao Paulo / Vinhedo	Human	1	197248	33.00	Lesion swab	IIb B.1

45	hMpxV/Georgia/NCDC-001/2022	EPI_ISL_13331598	Asia / Georgia / Tbilisi	Human	1	197434	32.97	Papular Swab	IIb B.1
46	hMpxV/Spain/AN-HUCSC-00001/2022	EPI_ISL_13339105	Europe / Spain / Andalusia	Human	1	197208	33.00	Not Available	IIb B.1.3
47	hMpxV/Brazil/RS-IAL-05/2022	EPI_ISL_13343697	South America / Brazil / Rio Grande do Sul / Porto Alegre	Human	1	197275	32.99	Lesion swab	IIb B.1
48	hMpxV/Brazil/SP-IAL-06/2022	EPI_ISL_13343718	South America / Brazil / Sao Paulo / Indaiatuba	Human	1	197264	32.99	Lesion swab	IIb B.1.10
49	hMpxV/Spain/CT-HUVH-60425/2022	EPI_ISL_13363142	Europe / Spain / Catalunya	Human	1	197181	33.00	Not Available	IIb B.1
50	hMpxV/Hungary/NBL-003/2022	EPI_ISL_13374487	Europe / Hungary / Budapest	Human	1	197237	32.99	Not Available	IIb B.1
51	hMpxV/Scotland/CVR-1a/2022	EPI_ISL_13409177	Europe / United Kingdom / Scotland	Human	1	197131	33.01	Not Available	IIb B.1.1
52	hMpxV/Scotland/CVR-1b/2022	EPI_ISL_13409178	Europe / United Kingdom / Scotland	Human	1	197131	33.01	Not Available	IIb B.1

53	hMpxV/Scotland/CVR-1e/2022	EPI_ISL_13409181	Europe / United Kingdom / Scotland	Human	1	197131	33.01	Not Available	IIb B.1
54	hMpxV/Brazil/SP-IAL-07/2022	EPI_ISL_13436658	South America / Brazil / Sao Paulo / Sao Paulo	Human	1	197309	32.99	Lesion swab	IIb B.1.12
55	hMpxV/Brazil/SP-IAL-08/2022	EPI_ISL_13436792	South America / Brazil / Sao Paulo / Indaiatuba	Human	1	197347	32.98	Lesion swab	IIb B.1.10
56	hMpxV/Brazil/SP-IAL-09/2022	EPI_ISL_13437056	South America / Brazil / Sao Paulo / Sao Bernado do Campo	Human	1	197347	32.98	Lesion swab	IIb B.1
57	hMpxV/Spain/MD-HULP-8887/2022	EPI_ISL_13449965	Europe / Spain / Madrid	Human	1	197185	33.00	Vesicle	IIb B.1
58	hMpxV/Brazil/SP-IAL-10/2022	EPI_ISL_13459346	South America / Brazil / Sao Paulo / Sao Paulo	Human	1	197347	32.98	Lesion swab	IIb B.1
59	hMpxV/Brazil/SP-IAL-11/2022	EPI_ISL_13459347	South America / Brazil / Sao	Human	1	197347	32.98	Lesion swab	IIb B.1.1

			Paulo / Sao Paulo						
60	hMpxV/Brazil/SP-IAL-12/2022	EPI_ISL_13459482	South America / Brazil / Sao Paulo / Sao Paulo	Human	1	197334	32.98	Croast	IIb B.1.1
61	hMpxV/Italy/PIE-OAS-02530345/2022	EPI_ISL_13502582	Europe / Italy / Piedmont	Human	1	197230	33.00	Vesicular swab	IIb B.1
62	hMpxV/Brazil/SP-IAL-15/2022	EPI_ISL_13508471	South America / Brazil / Sao Paulo / Sao Paulo	Human	1	197348	32.98	Lesion swab	IIb B.1.1
63	hMpxV/Peru/LIM-INS-001/2022	EPI_ISL_13530881	South America / Peru / Lima	Human	1	197200	33.00	Lesion swab	IIb B.1.6
64	hMpxV/Belgium/UZ_REGA-3/2022	EPI_ISL_13537923	Europe / Belgium	Human	1	197966	32.93	Not Available	IIb B.1
65	hMpxV/Belgium/UZ_REGA-4/2022	EPI_ISL_13537924	Europe / Belgium	Human	1	197970	32.93	Not Available	IIb B.1
66	hMpxV/Belgium/UZ_REGA-5/2022	EPI_ISL_13537925	Europe / Belgium	Human	1	197631	32.95	Not Available	IIb B.1
67	hMpxV/Belgium/UZ_REGA-6/2022	EPI_ISL_13537926	Europe / Belgium	Human	1	197908	32.94	Not Available	IIb B.1

68	hMpxV/Austria/MUW_1527495/2022	EPI_ISL_13573943	Europe / Austria / Vorarlberg	Human	1	197193	33.00	Lesion swab	IIb B.1.1
69	hMpxV/Germany/un-UMR-124757/2022	EPI_ISL_13584854	Europe / Germany	Human	1	197304	32.99	Swab	IIb B.1.1
70	hMpxV/Germany/un-UMR-124759/2022	EPI_ISL_13586184	Europe / Germany	Human	1	197241	33.00	Swab	IIb B.1.1
71	hMpxV/Mexico/NLE-UANL-001/2022	EPI_ISL_13607904	North America / Mexico / Nuevo Leon	Human	1	197584	32.96	Not Available	IIb B.1.7
72	hMpxV/Mexico/CMX-InDRE-IBT-001/2022	EPI_ISL_13624509	North America / Mexico / Mexico City	Human	1	197005	33.01	Scab	IIb B.1
73	hMpxV/Taiwan/CVDCDC-110-231642/2022	EPI_ISL_13632071	Asia / Taiwan / Taipei	Human	1	197315	32.99	Not Available	IIb B.1.5
74	hMpxV/Peru/LIM-INS-003/2022	EPI_ISL_13651349	South America / Peru / Lima	Human	1	197193	33.00	Lesion swab	IIb B.1.6
75	hMpxV/Peru/LIM-INS-004/2022	EPI_ISL_13651350	South America / Peru / Lima	Human	1	197193	33.00	Lesion swab	IIb B.1
76	hMpxV/Brazil/SP-IAL-17/2022	EPI_ISL_13705407	South America / Brazil / Sao Paulo / Sao Paulo	Human	1	197205	33.00	Lesion swab	IIb B.1.9

77	hMpxV/Netherlands/NH-AUMC-0001/2022	EPI_ISL_13728303	Europe / Netherlands / Noord-Holland / Amsterdam	Human	1	197008	33.02	Vesicle fluid	IIb B.1
78	hMpxV/Brazil/SP-IAL-18/2022	EPI_ISL_13732932	South America / Brazil / Sao Paulo / Sao Paulo	Human	1	197205	33.00	Lesion swab	IIb B.1.1
79	hMpxV/Germany/un-RKI-106/2022	EPI_ISL_13734241	Europe / Germany	Human	1	197139	33.01	Swab	IIb B.1.1
80	hMpxV/Germany/un-RKI-108/2022	EPI_ISL_13734243	Europe / Germany	Human	1	197140	33.01	Swab	IIb B.1.1
81	hMpxV/Germany/un-RKI-109/2022	EPI_ISL_13734244	Europe / Germany	Human	1	197140	33.01	Swab	IIb B.1.2
82	hMpxV/Germany/un-RKI-110/2022	EPI_ISL_13734245	Europe / Germany	Human	1	197139	33.01	Swab	IIb B.1
83	hMpxV/Germany/un-RKI-112/2022	EPI_ISL_13734247	Europe / Germany	Human	1	197139	33.01	Swab	IIb B.1
84	hMpxV/Germany/un-RKI-114/2022	EPI_ISL_13734249	Europe / Germany	Human	1	197139	33.01	Swab	IIb B.1
85	hMpxV/Germany/un-RKI-119/2022	EPI_ISL_13734251	Europe / Germany	Human	1	197139	33.01	Swab	IIb B.1.2

86	hMpxV/Germany/un-RKI-121/2022	EPI_ISL_13734253	Europe / Germany	Human	1	197139	33.01	Swab	IIb B.1
87	hMpxV/Germany/un-RKI-124/2022	EPI_ISL_13734256	Europe / Germany	Human	1	197140	33.01	Swab	IIb B.1.2
88	hMpxV/Germany/un-RKI-125/2022	EPI_ISL_13734257	Europe / Germany	Human	1	197139	33.01	Swab	IIb B.1.1
89	hMpxV/Germany/un-RKI-128/2022	EPI_ISL_13734260	Europe / Germany	Human	1	197139	33.01	Swab	IIb B.1
90	hMpxV/Germany/un-RKI-130/2022	EPI_ISL_13734262	Europe / Germany	Human	1	197139	33.01	Swab	IIb B.1.1
91	hMpxV/Germany/un-RKI-131/2022	EPI_ISL_13734263	Europe / Germany	Human	1	197140	33.01	Swab	IIb B.1
92	hMpxV/Germany/un-RKI-133/2022	EPI_ISL_13734265	Europe / Germany	Human	1	197139	33.01	Swab	IIb B.1.7
93	hMpxV/Germany/un-RKI-134/2022	EPI_ISL_13734266	Europe / Germany	Human	1	197140	33.01	Swab	IIb B.1.8
94	hMpxV/Germany/un-RKI-136/2022	EPI_ISL_13734268	Europe / Germany	Human	1	197139	33.01	Swab	IIb B.1
95	hMpxV/USA/CA-CDC-002/2022	EPI_ISL_13734270	North America / USA / California	Human	1	197767	32.95	Not Available	IIb B.1

96	hMpxV/USA/NY-CDC-002/2022	EPI_ISL_13744897	North America / USA / New York	Human	1	197771	32.95	Not Available	IIb B.1.14
97	hMpxV/USA/NY-CDC-003/2022	EPI_ISL_13744898	North America / USA / New York	Human	1	197742	32.96	Not Available	IIb B.1
98	hMpxV/USA/IL-CDC-001/2022	EPI_ISL_13744899	North America / USA / Illinois	Human	1	197742	32.96	Not Available	IIb B.1
99	hMpxV/USA/NY-CDC-004/2022	EPI_ISL_13744900	North America / USA / New York	Human	1	197764	32.95	Not Available	IIb B.1.14
100	hMpxV/USA/NY-CDC-005/2022	EPI_ISL_13744901	North America / USA / New York	Human	1	197794	32.95	Not Available	IIb B.1.1
101	hMpxV/Germany/un-RKI-160/2022	EPI_ISL_13744905	Europe / Germany	Human	1	197139	33.01	swab	IIb B.1.2
102	hMpxV/Germany/un-RKI-139/2022	EPI_ISL_13744906	Europe / Germany	Human	1	197139	33.01	swab	IIb B.1
103	hMpxV/Germany/un-RKI-165/2022	EPI_ISL_13744907	Europe / Germany	Human	1	197139	33.01	swab	IIb B.1.7
104	hMpxV/Germany/un-RKI-138/2022	EPI_ISL_13744909	Europe / Germany	Human	1	197139	33.01	swab	IIb B.1

105	hMpxV/Germany/un-RKI-141/2022	EPI_ISL_13744910	Europe / Germany	Human	1	197139	33.01	swab	IIb B.1.2
106	hMpxV/Germany/un-RKI-144/2022	EPI_ISL_13744913	Europe / Germany	Human	1	197139	33.01	swab	IIb B.1
107	hMpxV/Germany/un-RKI-146/2022	EPI_ISL_13744914	Europe / Germany	Human	1	197139	33.01	swab	IIb B.1
108	hMpxV/Germany/un-RKI-147/2022	EPI_ISL_13744915	Europe / Germany	Human	1	197139	33.01	swab	IIb B.1
109	hMpxV/Germany/un-RKI-148/2022	EPI_ISL_13744916	Europe / Germany	Human	1	197137	33.01	swab	IIb B.1
110	hMpxV/Germany/un-RKI-149/2022	EPI_ISL_13744917	Europe / Germany	Human	1	197139	33.01	swab	IIb B.1
111	hMpxV/Germany/un-RKI-150/2022	EPI_ISL_13744918	Europe / Germany	Human	1	197137	33.01	swab	IIb B.1
112	hMpxV/Germany/un-RKI-151/2022	EPI_ISL_13744919	Europe / Germany	Human	1	197139	33.01	swab	IIb B.1
113	hMpxV/Germany/un-RKI-153/2022	EPI_ISL_13744921	Europe / Germany	Human	1	197139	33.01	swab	IIb B.1.2
114	hMpxV/Germany/un-RKI-154/2022	EPI_ISL_13744922	Europe / Germany	Human	1	197139	33.01	swab	IIb B.1.1
115	hMpxV/Germany/un-RKI-155/2022	EPI_ISL_13744923	Europe / Germany	Human	1	197139	33.01	swab	IIb B.1.14

116	hMpxV/Germany/un-RKI-156/2022	EPI_ISL_13744924	Europe / Germany	Human	1	197139	33.01	swab	IIb B.1.5
117	hMpxV/Germany/un-RKI-157/2022	EPI_ISL_13744925	Europe / Germany	Human	1	197139	33.01	swab	IIb B.1.7
118	hMpxV/Germany/un-RKI-159/2022	EPI_ISL_13744927	Europe / Germany	Human	1	197139	33.01	swab	IIb B.1.5
119	hMpxV/Germany/un-RKI-161/2022	EPI_ISL_13744928	Europe / Germany	Human	1	197139	33.01	swab	IIb B.1
120	hMpxV/Germany/un-RKI-162/2022	EPI_ISL_13744929	Europe / Germany	Human	1	197139	33.01	swab	IIb B.1.5
121	hMpxV/Germany/un-RKI-163/2022	EPI_ISL_13744930	Europe / Germany	Human	1	197139	33.01	swab	IIb B.1.1
122	hMpxV/Germany/un-RKI-164/2022	EPI_ISL_13744931	Europe / Germany	Human	1	197139	33.01	swab	IIb B.1.8
123	hMpxV/Peru/LIM-INS-005/2022	EPI_ISL_13833194	South America / Peru / Lima	Human	1	197193	33.00	Lesion swab	IIb B.1.6
124	hMpxV/Peru/LIM-INS-008/2022	EPI_ISL_13833197	South America / Peru / Lima	Human	1	197194	33.00	Lesion swab	IIb B.1.6
125	hMpxV/Austria/MUW_1531254/2022	EPI_ISL_13842548	Europe / Austria / Vienna	Human	1	197193	33.00	Pustule swab	IIb B.1

126	hMpxV/Germany/BE-ChVir28154/2022	EPI_ISL_138 89436	Europe / Germany / Berlin	Human	1	197335	32.99	Not Available	IIb B.1
127	hMpxV/Germany/BE-ChVir28689/2022	EPI_ISL_138 89439	Europe / Germany / Berlin	Human	1	197335	32.99	Not Available	IIb B.1
128	hMpxV/Germany/BE-ChVir28707/2022	EPI_ISL_138 89440	Europe / Germany / Berlin	Human	1	197343	32.99	Not Available	IIb B.1
129	hMpxV/Germany/BE-ChVir28136/2022	EPI_ISL_138 89442	Europe / Germany / Berlin	Human	1	197431	32.97	Not Available	IIb B.1.14
130	hMpxV/Germany/BE-ChVir28581/2022	EPI_ISL_138 89448	Europe / Germany / Berlin	Human	1	197328	32.99	Not Available	IIb B.1.1
131	hMpxV/Germany/BE-ChVir28604/2022	EPI_ISL_138 89590	Europe / Germany / Berlin	Human	1	197337	32.99	Not Available	IIb B.1.7
132	hMpxV/Germany/BE-ChVir28152/2022	EPI_ISL_138 89660	Europe / Germany / Berlin	Human	1	197338	32.99	Not Available	IIb B.1.1
133	hMpxV/Germany/BE-ChVir28456/2022	EPI_ISL_138 89977	Europe / Germany / Berlin	Human	1	197346	32.99	Not Available	IIb B.1

134	hMpxV/Germany/BE-ChVir28703/2022	EPI_ISL_138 90408	Europe / Germany / Berlin	Human	1	197338	32.99	Not Available	IIb B.1
135	hMpxV/Germany/BE-ChVir28633/2022	EPI_ISL_138 90465	Europe / Germany / Berlin	Human	1	197325	32.99	Not Available	IIb B.1.2
136	hMpxV/Germany/BE-ChVir28599/2022	EPI_ISL_138 90468	Europe / Germany / Berlin	Human	1	197343	32.99	Not Available	IIb B.1.1
137	hMpxV/Germany/BE-ChVir28682/2022	EPI_ISL_138 90469	Europe / Germany / Berlin	Human	1	197347	32.99	Not Available	IIb B.1
138	hMpxV/Germany/BE-ChVir28389/2022	EPI_ISL_138 90482	Europe / Germany / Berlin	Human	1	196039	32.97	Not Available	IIb B.1.1
139	hMpxV/Germany/un-RKI169/2022	EPI_ISL_139 08346	Europe / Germany	Human	1	197140	33.01	swab	IIb B.1.2
140	hMpxV/Germany/un-RKI183/2022	EPI_ISL_139 08349	Europe / Germany	Human	1	197139	33.01	swab	IIb B.1.1
141	hMpxV/Germany/un-RKI167/2022	EPI_ISL_139 08351	Europe / Germany	Human	1	197140	33.01	swab	IIb B.1.2
142	hMpxV/Germany/un-RKI168/2022	EPI_ISL_139 08352	Europe / Germany	Human	1	197140	33.01	swab	IIb B.1

143	hMpxV/Germany/un-RKI171/2022	EPI_ISL_13908354	Europe / Germany	Human	1	197140	33.01	swab	IIb B.1
144	hMpxV/Germany/un-RKI172/2022	EPI_ISL_13908355	Europe / Germany	Human	1	197139	33.01	swab	IIb B.1
145	hMpxV/Germany/un-RKI176/2022	EPI_ISL_13908359	Europe / Germany	Human	1	197141	33.01	swab	IIb B.1
146	hMpxV/Germany/un-RKI177/2022	EPI_ISL_13908360	Europe / Germany	Human	1	197140	33.01	swab	IIb B.1.8
147	hMpxV/India/KL-ICMR-16-5316-553/2022	EPI_ISL_13953610	Asia / India / Kerala	Human	1	197120	33.02	Base of Blister Fluid	IIb A.2.1
148	hMpxV/USA/CA-CDPH-000002/2022	EPI_ISL_13993735	North America / USA / California	Human	1	197205	33.00	Not Available	IIb B.1.2
149	hMpxV/USA/CA-CDPH-000005/2022	EPI_ISL_13993738	North America / USA / California	Human	1	197205	33.00	Not Available	IIb B.1
150	hMpxV/USA/CA-CDPH-000006/2022	EPI_ISL_13993739	North America / USA / California	Human	1	197205	33.00	Not Available	IIb B.1
151	hMpxV/USA/WA-UW-0015/2022	EPI_ISL_14033207	North America / USA / Washington	Human	1	197176	33.00	Not Available	IIb B.1.1

152	hMpxV/USA/WA-UW-0022/2022	EPI_ISL_14033212	North America / USA / Washington	Human	1	197176	33.00	Not Available	IIb B.1
153	hMpxV/India/KL-ICMR-16-5316-570-P1/2022	EPI_ISL_14049244	Asia / India / Kerala	Human	1	197198	33.02	Base of Blister Fluid	IIb A.2.1
154	hMpxV/Brazil/SP-IAL-22/2022	EPI_ISL_14070493	South America / Brazil / Sao Paulo / Sao Paulo	Human	1	197198	33.00	Lesion swab	IIb B.1
155	hMpxV/Brazil/SP-IAL-23/2022	EPI_ISL_14070852	South America / Brazil / Sao Paulo / Sao Paulo	Human	1	197205	33.00	Lesion swab	IIb B.1
156	hMpxV/Brazil/SP-IAL-24/2022	EPI_ISL_14070854	South America / Brazil / Sao Paulo / Sao Paulo	Human	1	197149	33.00	Lesion swab	IIb B.1.1
157	hMpxV/Brazil/SP-IAL-25/2022	EPI_ISL_14070855	South America / Brazil / Sao Paulo / Sao Paulo	Human	1	197171	33.00	Lesion swab	IIb B.1.1
158	hMpxV/Austria/MUW-1533374/2022	EPI_ISL_14166709	Europe / Austria / Vienna	Human	1	197179	33.00	Not Available	IIb B.1

159	hMpxV/Austria/MUW-1534823/2022	EPI_ISL_14167248	Europe / Austria / Vienna	Human	1	197200	33.00	Lesion swab	IIb B.1
160	hMpxV/Austria/MUW-1531848/2022	EPI_ISL_14167573	Europe / Austria / Vienna	Human	1	197182	33.00	Lesion swab	IIb B.1
161	hMpxV/Austria/MUW-1532243/2022	EPI_ISL_14167574	Europe / Austria / Vienna	Human	1	197190	33.00	Genital Swab	IIb B.1
162	hMpxV/Austria/MUW-1536480/2022	EPI_ISL_14167575	Europe / Austria / Vienna	Human	1	197201	33.00	Lesion swab	IIb B.1
163	hMpxV/Spain/un-ISCIII-353_R/2022	EPI_ISL_14181948	Europe / Spain	Human	1	197375	32.99	Not Available	IIb B.1
164	hMpxV/Spain/un-ISCIII-351_R/2022	EPI_ISL_14181949	Europe / Spain	Human	1	197358	32.99	Not Available	IIb B.1
165	hMpxV/Spain/un-ISCIII-345_R/2022	EPI_ISL_14181951	Europe / Spain	Human	1	197225	33.00	Not Available	IIb B.1
166	hMpxV/Spain/un-ISCIII-350_R/2022	EPI_ISL_14181952	Europe / Spain	Human	1	197290	33.00	Not Available	IIb B.1
167	hMpxV/Spain/un-ISCIII-352_R/2022	EPI_ISL_14181953	Europe / Spain	Human	1	197314	32.99	Not Available	IIb B.1.8

168	hMpxV/Spain/un-ISCIII-349_R/2022	EPI_ISL_14181954	Europe / Spain	Human	1	197374	32.99	Not Available	IIb B.1
169	hMpxV/Spain/un-ISCIII-399/2022	EPI_ISL_14181955	Europe / Spain	Human	1	197342	32.99	Not Available	IIb B.1
170	hMpxV/Spain/un-ISCIII-453/2022	EPI_ISL_14181956	Europe / Spain	Human	1	197162	33.00	Not Available	IIb B.1
171	hMpxV/Spain/un-ISCIII-403/2022	EPI_ISL_14181958	Europe / Spain	Human	1	197293	32.99	Not Available	IIb B.1
172	hMpxV/Peru/LIM-INS-012/2022	EPI_ISL_14207727	South America / Peru / Lima	Human	1	197193	33.00	Lesion swab	IIb B.1.6
173	hMpxV/Peru/LIM-INS-014/2022	EPI_ISL_14207729	South America / Peru / Lima	Human	1	197193	33.00	Lesion swab	IIb B.1.6
174	hMpxV/Peru/LIM-INS-015/2022	EPI_ISL_14207730	South America / Peru / Lima	Human	1	197193	33.00	Lesion swab	IIb B.1.6
175	hMpxV/Peru/LIM-INS-016/2022	EPI_ISL_14207731	South America / Peru / Lima	Human	1	197193	33.00	Lesion swab	IIb B.1.6
176	hMpxV/Peru/LIM-INS-018/2022	EPI_ISL_14207733	South America / Peru / Lima	Human	1	197193	33.00	Lesion swab	IIb B.1.6
177	hMpxV/Peru/LIM-INS-020/2022	EPI_ISL_14207735	South America / Peru / Lima	Human	1	197193	33.00	Lesion swab	IIb B.1.6
178	hMpxV/Peru/LAL-INS-022/2022	EPI_ISL_14207737	South America / Peru / La Libertad	Human	1	197194	33.00	Lesion swab	IIb B.1.6

179	hMpxV/Peru/LAL-INS-025/2022	EPI_ISL_14207740	South America / Peru / La Libertad	Human	1	197193	33.00	Lesion swab	IIb B.1.6
180	hMpxV/Peru/LIM-INS-026/2022	EPI_ISL_14207741	South America / Peru / Lima	Human	1	197193	33.00	Lesion swab	IIb B.1.6
181	hMpxV/USA/un-UW-0024/2022	EPI_ISL_14216746	North America / USA	Human	1	197174	33.00	Not Available	IIb B.1
182	hMpxV/USA/un-UW-0026/2022	EPI_ISL_14216750	North America / USA	Human	1	197176	33.00	Not Available	IIb B.1
183	hMpxV/USA/un-UW-0027/2022	EPI_ISL_14216752	North America / USA	Human	1	197176	33.00	Not Available	IIb B.1
184	hMpxV/USA/un-UW-0028/2022	EPI_ISL_14216753	North America / USA	Human	1	197176	33.00	Not Available	IIb B.1
185	hMpxV/USA/un-UW-0029/2022	EPI_ISL_14216754	North America / USA	Human	1	197176	33.00	Not Available	IIb B.1.2
186	hMpxV/USA/un-UW-0030/2022	EPI_ISL_14216755	North America / USA	Human	1	197174	33.00	Not Available	IIb B.1.1
187	hMpxV/USA/un-UW-0032/2022	EPI_ISL_14216757	North America / USA	Human	1	197176	33.00	Not Available	IIb B.1
188	hMpxV/USA/un-UW-0033/2022	EPI_ISL_14216758	North America / USA	Human	1	197176	33.00	Not Available	IIb B.1.3
189	hMpxV/USA/un-UW-0035/2022	EPI_ISL_14216761	North America / USA	Human	1	197176	33.00	Not Available	IIb B.1.13

190	hMpxV/USA/un-UW-0036/2022	EPI_ISL_14216762	North America / USA	Human	1	197176	33.00	Not Available	IIb B.1.1
191	hMpxV/USA/un-UW-0037/2022	EPI_ISL_14216764	North America / USA	Human	1	197176	33.00	Not Available	IIb B.1.1
192	hMpxV/USA/un-UW-0039/2022	EPI_ISL_14216769	North America / USA	Human	1	197180	33.00	Not Available	IIb B.1.11
193	hMpxV/Chile/RM-ISP-75625/2022	EPI_ISL_14224334	South America / Chile / Region Metropolitana de Santiago	Human	1	197205	33.00	Not Available	IIb B.1
194	hMpxV/USA/CA-CDC-PRB-006/2022	EPI_ISL_14244556	North America / USA / California	Human	1	197776	32.95	Lesion swab	IIb B.1.3
195	hMpxV/USA/UW-WA-0043/2022	EPI_ISL_14315315	North America / USA / Washington	Human	1	197176	33.00	Not Available	IIb B.1.13
196	hMpxV/USA/UW-WA-0045/2022	EPI_ISL_14315317	North America / USA / Washington	Human	1	197168	33.00	Not Available	IIb B.1
197	hMpxV/USA/UW-WA-0046/2022	EPI_ISL_14315318	North America / USA / Washington	Human	1	197176	33.00	Not Available	IIb B.1

198	hMpxV/USA/UW-WA-0047/2022	EPI_ISL_14315319	North America / USA / Washington	Human	1	197178	33.00	Not Available	IIb B.1.1
199	hMpxV/USA/UW-WA-0049/2022	EPI_ISL_14315320	North America / USA / Washington	Human	1	197176	33.00	Not Available	IIb B.1.2
200	hMpxV/USA/UW-WA-0051/2022	EPI_ISL_14315322	North America / USA / Washington	Human	1	197174	33.00	Not Available	IIb B.1.1
201	hMpxV/USA/UW-WA-0052/2022	EPI_ISL_14315323	North America / USA / Washington	Human	1	197174	33.00	Not Available	IIb B.1.3
202	hMpxV/USA/NE-EAOH-75/2022	EPI_ISL_14326638	North America / USA / Nebraska	Human	1	197176	33.00	Not Available	IIb B.1.12
203	hMpxV/USA/NE-EAOH-63/2022	EPI_ISL_14326639	North America / USA / Nebraska	Human	1	197176	33.00	Not Available	IIb B.1.3
204	hMpxV/USA/NE-EAOH-48/2022	EPI_ISL_14326640	North America / USA / Nebraska	Human	1	197176	33.00	Not Available	IIb B.1.8
205	hMpxV/USA/NE-EAOH-44/2022	EPI_ISL_14326641	North America / USA / Nebraska	Human	1	197176	33.00	Not Available	IIb B.1

206	hMpxV/USA/NE-EAOH-43/2022	EPI_ISL_14326642	North America / USA / Nebraska	Human	1	197176	33.00	Not Available	IIb B.1.3
207	hMpxV/USA/NE-EAOH-31/2022	EPI_ISL_14326643	North America / USA / Nebraska	Human	1	197176	33.00	Not Available	IIb B.1.12
208	hMpxV/USA/NE-EAOH-25/2022	EPI_ISL_14326644	North America / USA / Nebraska	Human	1	197176	33.00	Not Available	IIb B.1.12
209	hMpxV/USA/WA-UW-0056/2022	EPI_ISL_14355206	North America / USA / Washington	Human	1	197176	33.00	Not Available	IIb B.1
210	hMpxV/USA/WA-UW-0058/2022	EPI_ISL_14355207	North America / USA / Washington	Human	1	197176	33.00	Not Available	IIb B.1
211	hMpxV/USA/WA-UW-0059/2022	EPI_ISL_14355208	North America / USA / Washington	Human	1	197176	33.00	Not Available	IIb B.1.3
212	hMpxV/USA/WA-UW-0061/2022	EPI_ISL_14355210	North America / USA / Washington	Human	1	197174	33.00	Not Available	IIb B.1.1
213	hMpxV/USA/WA-UW-0062/2022	EPI_ISL_14355211	North America / USA / Washington	Human	1	197176	33.00	Not Available	IIb B.1.2

214	hMpxV/Japan/TKY-TMIPH-0091/2022	EPI_ISL_14394060	Asia / Japan / Tokyo	Human	1	197141	33.01	Swab	IIb B.1
215	hMpxV/United Kingdom/UKHSA-8/2022	EPI_ISL_14439713	Europe / United Kingdom	Human	1	197207	33.00	Not Available	IIb B.1
216	hMpxV/United Kingdom/UKHSA-9/2022	EPI_ISL_14439714	Europe / United Kingdom	Human	1	197207	33.00	Not Available	IIb B.1
217	hMpxV/United Kingdom/UKHSA-10/2022	EPI_ISL_14439715	Europe / United Kingdom	Human	1	197205	33.00	Not Available	IIb B.1.7
218	hMpxV/United Kingdom/UKHSA-11/2022	EPI_ISL_14439716	Europe / United Kingdom	Human	1	197202	33.00	Not Available	IIb B.1.7
219	hMpxV/United Kingdom/UKHSA-13/2022	EPI_ISL_14439718	Europe / United Kingdom	Human	1	197209	33.00	Not Available	IIb B.1.2
220	hMpxV/United Kingdom/UKHSA-15/2022	EPI_ISL_14439720	Europe / United Kingdom	Human	1	197205	33.00	Not Available	IIb B.1.7
221	hMpxV/United Kingdom/UKHSA-17/2022	EPI_ISL_14439722	Europe / United Kingdom	Human	1	197205	33.00	Not Available	IIb B.1.7

222	hMpxV/United Kingdom/UKHSA-19/2022	EPI_ISL_14439724	Europe / United Kingdom	Human	1	197205	33.00	Not Available	IIb B.1
223	hMpxV/United Kingdom/UKHSA-21/2022	EPI_ISL_14439726	Europe / United Kingdom	Human	1	197205	33.00	Not Available	IIb B.1.7
224	hMpxV/United Kingdom/UKHSA-25/2022	EPI_ISL_14439730	Europe / United Kingdom	Human	1	197205	33.00	Not Available	IIb B.1
225	hMpxV/United Kingdom/UKHSA-26/2022	EPI_ISL_14439731	Europe / United Kingdom	Human	1	197203	33.00	Not Available	IIb B.1.2
226	hMpxV/United Kingdom/UKHSA-29/2022	EPI_ISL_14439734	Europe / United Kingdom	Human	1	197205	33.00	Not Available	IIb B.1.2
227	hMpxV/United Kingdom/UKHSA-31/2022	EPI_ISL_14439736	Europe / United Kingdom	Human	1	197205	33.00	Not Available	IIb B.1.10
228	hMpxV/United Kingdom/UKHSA-32/2022	EPI_ISL_14439737	Europe / United Kingdom	Human	1	197207	33.00	Not Available	IIb B.1
229	hMpxV/United Kingdom/UKHSA-34/2022	EPI_ISL_14439739	Europe / United Kingdom	Human	1	197209	33.00	Not Available	IIb B.1.7

230	hMpxV/United Kingdom/UKHSA-36/2022	EPI_ISL_14439741	Europe / United Kingdom	Human	1	197207	33.00	Not Available	IIb B.1
231	hMpxV/United Kingdom/UKHSA-37/2022	EPI_ISL_14439742	Europe / United Kingdom	Human	1	197205	33.00	Not Available	IIb B.1.7
232	hMpxV/United Kingdom/UKHSA-40/2022	EPI_ISL_14439745	Europe / United Kingdom	Human	1	197205	33.00	Not Available	IIb B.1.7
233	hMpxV/United Kingdom/UKHSA-41/2022	EPI_ISL_14439746	Europe / United Kingdom	Human	1	197205	33.00	Not Available	IIb B.1.7
234	hMpxV/United Kingdom/UKHSA-42/2022	EPI_ISL_14439747	Europe / United Kingdom	Human	1	197205	33.00	Not Available	IIb B.1
235	hMpxV/United Kingdom/UKHSA-44/2022	EPI_ISL_14439749	Europe / United Kingdom	Human	1	197205	33.00	Not Available	IIb B.1
236	hMpxV/United Kingdom/UKHSA-47/2022	EPI_ISL_14439752	Europe / United Kingdom	Human	1	197205	33.00	Not Available	IIb B.1
237	hMpxV/United Kingdom/UKHSA-48/2022	EPI_ISL_14439753	Europe / United Kingdom	Human	1	197205	33.00	Not Available	IIb B.1

238	hMpxV/United Kingdom/UKHSA-53/2022	EPI_ISL_144 39758	Europe / United Kingdom	Human	1	197205	33.00	Not Available	IIb B.1
239	hMpxV/United Kingdom/UKHSA-54/2022	EPI_ISL_144 39759	Europe / United Kingdom	Human	1	197205	33.00	Not Available	IIb B.1
240	hMpxV/United Kingdom/UKHSA-55/2022	EPI_ISL_144 39760	Europe / United Kingdom	Human	1	197205	33.00	Not Available	IIb B.1
241	hMpxV/United Kingdom/UKHSA-57/2022	EPI_ISL_144 39762	Europe / United Kingdom	Human	1	197205	33.00	Not Available	IIb B.1
242	hMpxV/United Kingdom/UKHSA-59/2022	EPI_ISL_144 39764	Europe / United Kingdom	Human	1	197205	33.00	Not Available	IIb B.1.7
243	hMpxV/United Kingdom/UKHSA-61/2022	EPI_ISL_144 39766	Europe / United Kingdom	Human	1	197205	33.00	Not Available	IIb B.1.3
244	hMpxV/United Kingdom/UKHSA-62/2022	EPI_ISL_144 39767	Europe / United Kingdom	Human	1	197205	33.00	Not Available	IIb B.1.7
245	hMpxV/United Kingdom/UKHSA-63/2022	EPI_ISL_144 39768	Europe / United Kingdom	Human	1	197205	33.00	Not Available	IIb B.1

246	hMpxV/United Kingdom/UKHSA-64/2022	EPI_ISL_14439769	Europe / United Kingdom	Human	1	197205	33.00	Not Available	IIb B.1
247	hMpxV/United Kingdom/UKHSA-69/2022	EPI_ISL_14439774	Europe / United Kingdom	Human	1	197205	33.00	Not Available	IIb B.1
248	hMpxV/United Kingdom/UKHSA-70/2022	EPI_ISL_14439775	Europe / United Kingdom	Human	1	197205	33.00	Not Available	IIb B.1
249	hMpxV/United Kingdom/UKHSA-79/2022	EPI_ISL_14439784	Europe / United Kingdom	Human	1	197205	33.00	Not Available	IIb B.1
250	hMpxV/United Kingdom/UKHSA-80/2022	EPI_ISL_14439785	Europe / United Kingdom	Human	1	197205	33.00	Not Available	IIb B.1.2
251	hMpxV/Peru/LAL-INS-060/2022	EPI_ISL_14445101	South America / Peru / La Libertad	Human	1	197193	33.00	Lesion swab	IIb B.1.6
252	hMpxV/Peru/LIM-INS-030/2022	EPI_ISL_14445109	South America / Peru / Lima	Human	1	197193	33.00	Lesion swab	IIb B.1
253	hMpxV/Peru/LIM-INS-043/2022	EPI_ISL_14445120	South America / Peru / Lima	Human	1	197193	33.00	Lesion swab	IIb B.1.6
254	hMpxV/Peru/LIM-INS-045/2022	EPI_ISL_14445122	South America / Peru / Lima	Human	1	197193	33.00	Lesion swab	IIb B.1.6

255	hMpxV/Peru/LIM-INS-046/2022	EPI_ISL_14445123	South America / Peru / Lima	Human	1	197193	33.00	Lesion swab	IIb B.1.6
256	hMpxV/Peru/LIM-INS-047/2022	EPI_ISL_14445124	South America / Peru / Lima	Human	1	197193	33.00	Lesion swab	IIb B.1.6
257	hMpxV/Peru/LIM-INS-049/2022	EPI_ISL_14445126	South America / Peru / Lima	Human	1	197193	33.00	Lesion swab	IIb B.1
258	hMpxV/Peru/LIM-INS-050/2022	EPI_ISL_14445127	South America / Peru / Lima	Human	1	197193	33.00	Nasopharyngeal and Oropharyngeal swab	IIb B.1.6
259	hMpxV/Peru/LIM-INS-052/2022	EPI_ISL_14445128	South America / Peru / Lima	Human	1	197193	33.00	Lesion swab	IIb B.1.6
260	hMpxV/Peru/LIM-INS-053/2022	EPI_ISL_14445129	South America / Peru / Lima	Human	1	197193	33.00	Lesion swab	IIb B.1.6
261	hMpxV/Peru/LIM-INS-054/2022	EPI_ISL_14445130	South America / Peru / Lima	Human	1	197193	33.00	Lesion swab	IIb B.1
262	hMpxV/Peru/LIM-INS-057/2022	EPI_ISL_14445133	South America / Peru / Lima	Human	1	197193	33.00	Lesion swab	IIb B.1.6
263	hMpxV/Peru/LIM-INS-064/2022	EPI_ISL_14445138	South America / Peru / Lima	Human	1	197193	33.00	Lesion swab	IIb B.1.6
264	hMpxV/Peru/LIM-INS-065/2022	EPI_ISL_14445139	South America / Peru / Lima	Human	1	197193	33.00	Lesion swab	IIb B.1.6

265	hMpxV/Peru/LIM-INS-067/2022	EPI_ISL_14445141	South America / Peru / Lima	Human	1	197193	33.00	Nasopharyngeal and Oropharyngeal swab	IIb B.1.6
266	hMpxV/Peru/LIM-INS-068/2022	EPI_ISL_14445142	South America / Peru / Lima	Human	1	197200	33.00	Lesion swab	IIb B.1.6
267	hMpxV/Peru/LIM-INS-071/2022	EPI_ISL_14445144	South America / Peru / Lima	Human	1	197193	33.00	Lesion swab	IIb B.1.6
268	hMpxV/Germany/un-RKI-196/2022	EPI_ISL_14445158	Europe / Germany	Human	1	197139	33.01	Swab	IIb B.1.2
269	hMpxV/Germany/un-RKI-198/2022	EPI_ISL_14445159	Europe / Germany	Human	1	197139	33.01	Swab	IIb B.1
270	hMpxV/Germany/un-RKI-209/2022	EPI_ISL_14445163	Europe / Germany	Human	1	197139	33.01	Swab	IIb B.1
271	hMpxV/South Korea/KDCPA-001/2022	EPI_ISL_14494949	Asia / South Korea	Human	1	197200	33.00	Skin swab	IIb B.1.1
272	hMpxV/Germany/un-RKI235/2022	EPI_ISL_14515108	Europe / Germany	Human	1	197139	33.01	swab	IIb B.1.8
273	hMpxV/Germany/un-RKI242/2022	EPI_ISL_14515111	Europe / Germany	Human	1	197140	33.01	swab	IIb B.1
274	hMpxV/Germany/un-RKI250/2022	EPI_ISL_14515113	Europe / Germany	Human	1	197140	33.01	swab	IIb B.1

275	hMpxV/Germany/un-RKI187/2022	EPI_ISL_14515114	Europe / Germany	Human	1	197139	33.01	swab	IIb B.1.7
276	hMpxV/Germany/un-RKI190/2022	EPI_ISL_14515116	Europe / Germany	Human	1	197140	33.01	swab	IIb B.1.8
277	hMpxV/Germany/un-RKI191/2022	EPI_ISL_14515117	Europe / Germany	Human	1	197139	33.01	swab	IIb B.1
278	hMpxV/Germany/un-RKI192/2022	EPI_ISL_14515118	Europe / Germany	Human	1	197139	33.01	swab	IIb B.1
279	hMpxV/Germany/un-RKI193/2022	EPI_ISL_14515119	Europe / Germany	Human	1	197139	33.01	swab	IIb B.1.2
280	hMpxV/Germany/un-RKI194/2022	EPI_ISL_14515120	Europe / Germany	Human	1	197139	33.01	swab	IIb B.1
281	hMpxV/Germany/un-RKI195/2022	EPI_ISL_14515121	Europe / Germany	Human	1	197140	33.01	swab	IIb B.1
282	hMpxV/Germany/un-RKI199/2022	EPI_ISL_14515123	Europe / Germany	Human	1	197140	33.01	swab	IIb B.1
283	hMpxV/Germany/un-RKI201/2022	EPI_ISL_14515125	Europe / Germany	Human	1	197139	33.01	swab	IIb B.1
284	hMpxV/Germany/un-RKI205/2022	EPI_ISL_14515126	Europe / Germany	Human	1	197140	33.00	swab	IIb B.1.1
285	hMpxV/Germany/un-RKI210/2022	EPI_ISL_14515130	Europe / Germany	Human	1	197139	33.01	swab	IIb B.1

286	hMpxV/Germany/un-RKI215/2022	EPI_ISL_14515132	Europe / Germany	Human	1	197139	33.01	swab	IIb B.1.1
287	hMpxV/Germany/un-RKI220/2022	EPI_ISL_14515134	Europe / Germany	Human	1	197140	33.01	swab	IIb B.1.3
288	hMpxV/Germany/un-RKI222/2022	EPI_ISL_14515136	Europe / Germany	Human	1	197139	33.01	swab	IIb B.1.1
289	hMpxV/Germany/un-RKI232/2022	EPI_ISL_14515142	Europe / Germany	Human	1	197140	33.01	swab	IIb B.1
290	hMpxV/Germany/un-RKI236/2022	EPI_ISL_14515144	Europe / Germany	Human	1	197139	33.01	swab	IIb B.1
291	hMpxV/Germany/un-RKI239/2022	EPI_ISL_14515147	Europe / Germany	Human	1	197141	33.01	swab	IIb B.1
292	hMpxV/Germany/un-RKI244/2022	EPI_ISL_14515148	Europe / Germany	Human	1	197139	33.01	swab	IIb B.1
293	hMpxV/Germany/un-RKI247/2022	EPI_ISL_14515151	Europe / Germany	Human	1	197139	33.01	swab	IIb B.1.1
294	hMpxV/Germany/un-RKI252/2022	EPI_ISL_14515153	Europe / Germany	Human	1	197139	33.01	swab	IIb B.1
295	hMpxV/Germany/un-RKI255/2022	EPI_ISL_14515154	Europe / Germany	Human	1	197139	33.01	swab	IIb B.1
296	hMpxV/Germany/un-RKI269/2022	EPI_ISL_14515155	Europe / Germany	Human	1	197139	33.01	swab	IIb B.1

297	hMpxV/Germany/un-RKI253/2022	EPI_ISL_14515156	Europe / Germany	Human	1	197117	33.01	swab	IIb B.1.1
298	hMpxV/Germany/un-RKI251/2022	EPI_ISL_14515157	Europe / Germany	Human	1	197139	33.01	swab	IIb B.1.3
299	hMpxV/Germany/un-RKI254/2022	EPI_ISL_14515158	Europe / Germany	Human	1	197139	33.01	swab	IIb B.1.2
300	hMpxV/Germany/un-RKI256/2022	EPI_ISL_14515159	Europe / Germany	Human	1	197139	33.01	swab	IIb B.1
301	hMpxV/Germany/un-RKI259/2022	EPI_ISL_14515162	Europe / Germany	Human	1	197139	33.01	swab	IIb B.1.1
302	hMpxV/Germany/un-RKI262/2022	EPI_ISL_14515165	Europe / Germany	Human	1	197139	33.01	swab	IIb B.1
303	hMpxV/Germany/un-RKI264/2022	EPI_ISL_14515167	Europe / Germany	Human	1	197139	33.01	swab	IIb B.1
304	hMpxV/Germany/un-RKI265/2022	EPI_ISL_14515168	Europe / Germany	Human	1	197140	33.01	swab	IIb B.1
305	hMpxV/Germany/un-RKI267/2022	EPI_ISL_14515170	Europe / Germany	Human	1	197140	33.01	swab	IIb B.1
306	hMpxV/USA/WA-UW-0065/2022	EPI_ISL_14562479	North America / USA / Washington	Human	1	197176	33.00	Not Available	IIb B.1

307	hMpxV/USA/WA-UW-0069/2022	EPI_ISL_14562481	North America / USA / Washington	Human	1	197176	33.00	Not Available	IIb B.1
308	hMpxV/USA/WA-UW-0071/2022	EPI_ISL_14562483	North America / USA / Washington	Human	1	197176	33.00	Not Available	IIb B.1.2
309	hMpxV/USA/WA-UW-0075/2022	EPI_ISL_14562484	North America / USA / Washington	Human	1	197176	33.00	Not Available	IIb B.1
310	hMpxV/USA/WA-UW-0076/2022	EPI_ISL_14562485	North America / USA / Washington	Human	1	197178	33.00	Not Available	IIb B.1.11
311	hMpxV/USA/WA-UW-0078/2022	EPI_ISL_14562486	North America / USA / Washington	Human	1	197178	33.00	Not Available	IIb B.1.1
312	hMpxV/USA/WA-UW-0079/2022	EPI_ISL_14562487	North America / USA / Washington	Human	1	197176	33.00	Not Available	IIb B.1
313	hMpxV/USA/WA-UW-0080/2022	EPI_ISL_14562488	North America / USA / Washington	Human	1	197176	33.00	Not Available	IIb B.1
314	hMpxV/USA/WA-UW-0084/2022	EPI_ISL_14562490	North America / USA / Washington	Human	1	197168	33.00	Not Available	IIb B.1

315	hMpxV/USA/WA-UW-0088/2022	EPI_ISL_14562492	North America / USA / Washington	Human	1	197174	33.00	Not Available	IIb B.1.1
316	hMpxV/USA/WA-UW-0089/2022	EPI_ISL_14562493	North America / USA / Washington	Human	1	197176	33.00	Not Available	IIb B.1.3
317	hMpxV/USA/WA-UW-0090/2022	EPI_ISL_14562494	North America / USA / Washington	Human	1	197178	33.00	Not Available	IIb B.1.11
318	hMpxV/USA/WA-UW-0092/2022	EPI_ISL_14562496	North America / USA / Washington	Human	1	197176	33.00	Not Available	IIb B.1
319	hMpxV/USA/WA-UW-0094/2022	EPI_ISL_14562498	North America / USA / Washington	Human	1	197176	33.00	Not Available	IIb B.1
320	hMpxV/USA/WA-UW-0097/2022	EPI_ISL_14562500	North America / USA / Washington	Human	1	197176	33.00	Not Available	IIb B.1
321	hMpxV/USA/WA-UW-00101/2022	EPI_ISL_14562502	North America / USA / Washington	Human	1	197174	33.00	Not Available	IIb B.1
322	hMpxV/Germany/un-RKI274/2022	EPI_ISL_14562504	Europe / Germany	Human	1	197139	33.01	swab	IIb B.1.7

323	hMpxV/Germany/un-RKI276/2022	EPI_ISL_14562507	Europe / Germany	Human	1	197139	33.01	swab	IIb B.1
324	hMpxV/Germany/un-RKI280/2022	EPI_ISL_14562511	Europe / Germany	Human	1	197139	33.01	swab	IIb B.1
325	hMpxV/Germany/un-RKI281/2022	EPI_ISL_14562512	Europe / Germany	Human	1	197139	33.01	swab	IIb B.1.2
326	hMpxV/Brazil/SP-IAL-29/2022	EPI_ISL_14571433	South America / Brazil / Sao Paulo / Caraguatatuba	Human	1	196491	33.01	Lesion swab	IIb B.1.1
327	hMpxV/Brazil/SP-IAL-33/2022	EPI_ISL_14571442	South America / Brazil / Sao Paulo / Guaruja	Human	1	196496	33.01	Lesion swab	IIb B.1
328	hMpxV/Peru/LIM-INS-105/2022	EPI_ISL_14584283	South America / Peru / Lima	Human	1	197193	33.00	Lesion swab	IIb B.1.2
329	hMpxV/Peru/LIM-INS-089/2022	EPI_ISL_14584284	South America / Peru / Lima	Human	1	197197	33.00	Lesion swab	IIb B.1.6
330	hMpxV/Peru/LIM-INS-107/2022	EPI_ISL_14584286	South America / Peru / Lima	Human	1	197193	33.00	Lesion swab	IIb B.1.6
331	hMpxV/Peru/CAL-INS-095/2022	EPI_ISL_14584293	South America / Peru / Callao	Human	1	197193	33.00	Lesion swab	IIb B.1.6

332	hMpxV/Peru/LAL-INS-100/2022	EPI_ISL_14584302	South America / Peru / La Libertad	Human	1	197194	33.00	Lesion swab	IIb B.1.6
333	hMpxV/Peru/LIM-INS-103/2022	EPI_ISL_14584303	South America / Peru / Lima	Human	1	197193	33.00	Lesion swab	IIb B.1.6
334	hMpxV/Germany/un-RKI284/2022	EPI_ISL_14587554	Europe / Germany	Human	1	197140	33.01	Swab	IIb B.1.8
335	hMpxV/Germany/un-RKI285/2022	EPI_ISL_14587555	Europe / Germany	Human	1	197140	33.01	Swab	IIb B.1.2
336	hMpxV/Brazil/SP-IAL-35/2022	EPI_ISL_14622055	South America / Brazil / Sao Paulo / Sao Paulo	Human	1	196491	33.01	Lesion swab	IIb B.1.1
337	hMpxV/Brazil/SP-IAL-37/2022	EPI_ISL_14622705	South America / Brazil / Sao Paulo / Maua	Human	1	196606	33.00	Lesion swab	IIb B.1.1
338	hMpxV/Brazil/SP-IAL-38/2022	EPI_ISL_14622706	South America / Brazil / Sao Paulo / Bauru	Human	1	196715	33.01	Lesion swab	IIb B.1.1
339	hMpxV/Brazil/SP-IAL-39/2022	EPI_ISL_14622707	South America / Brazil / Sao Paulo / Sao Sebastiao	Human	1	196831	33.01	Lesion swab	IIb B.1.1

340	hMpxV/Brazil/RS-IAL-41/2022	EPI_ISL_14622953	South America / Brazil / Rio Grande do Sul / Viamao	Human	1	196481	33.00	Lesion swab	IIb B.1.1
341	hMpxV/Brazil/SP-IAL-42/2022	EPI_ISL_14622960	South America / Brazil / Sao Paulo / Patrocinio Paulista	Human	1	197137	33.01	Lesion swab	IIb B.1.9
342	hMpxV/Brazil/SP-IAL-43/2022	EPI_ISL_14623175	South America / Brazil / Sao Paulo / Ribeirao Preto	Human	1	196733	33.00	Lesion swab	IIb B.1.1
343	hMpxV/Brazil/SP-IAL-44/2022	EPI_ISL_14623523	South America / Brazil / Sao Paulo / Piracicaba	Human	1	196617	33.01	Lesion swab	IIb B.1.9
344	hMpxV/Brazil/SP-IAL-45/2022	EPI_ISL_14623704	South America / Brazil / Sao Paulo / Jundiai	Human	1	196742	33.01	Lesion swab	IIb B.1.1
345	hMpxV/Brazil/SP-IAL-46/2022	EPI_ISL_14624411	South America / Brazil / Sao Paulo / Atibaia	Human	1	196343	33.01	Lesion swab	IIb B.1.1
346	hMpxV/Brazil/SP-IAL-47/2022	EPI_ISL_14624610	South America / Brazil / Sao	Human	1	196489	33.01	Lesion swab	IIb B.1

			Paulo / Praia Grande						
347	hMpxV/Brazil/SP-IAL-49/2022	EPI_ISL_14624832	South America / Brazil / Sao Paulo / Guaruja	Human	1	196864	33.00	Lesion swab	IIb B.1.9
348	hMpxV/Brazil/SP-IAL-52/2022	EPI_ISL_14625157	South America / Brazil / Sao Paulo / Fartura	Human	1	196501	33.01	Lesion swab	IIb B.1.10
349	hMpxV/Brazil/PR-IAL-54/2022	EPI_ISL_14625230	South America / Brazil / Parana / Londrina	Human	1	196930	33.00	Lesion swab	IIb B.1.1
350	hMpxV/Brazil/PR-IAL-55/2022	EPI_ISL_14625256	South America / Brazil / Parana / Curitiba	Human	1	196813	33.00	Lesion swab	IIb B.1.1
351	hMpxV/USA/WA-UW-0105/2022	EPI_ISL_14699928	North America / USA / Washington	Human	1	197176	33.00	Not Available	IIb B.1
352	hMpxV/USA/WA-UW-0106/2022	EPI_ISL_14699929	North America / USA / Washington	Human	1	197176	33.00	Not Available	IIb B.1
353	hMpxV/USA/WA-UW-0108/2022	EPI_ISL_14699931	North America / USA / Washington	Human	1	197180	33.00	Not Available	IIb B.1.1

354	hMpxV/USA/WA-UW-0113/2022	EPI_ISL_14699935	North America / USA / Washington	Human	1	197177	33.00	Not Available	IIb B.1.2
355	hMpxV/USA/WA-UW-0116/2022	EPI_ISL_14699937	North America / USA / Washington	Human	1	197176	33.00	Not Available	IIb B.1
356	hMpxV/USA/WA-UW-0120/2022	EPI_ISL_14699939	North America / USA / Washington	Human	1	197176	33.00	Not Available	IIb B.1.13
357	hMpxV/USA/WA-UW-0125/2022	EPI_ISL_14699941	North America / USA / Washington	Human	1	197178	33.00	Not Available	IIb B.1.11
358	hMpxV/USA/WA-UW-0127/2022	EPI_ISL_14699943	North America / USA / Washington	Human	1	197178	33.00	Not Available	IIb B.1.1
359	hMpxV/USA/WA-UW-0130/2022	EPI_ISL_14699945	North America / USA / Washington	Human	1	197176	33.00	Not Available	IIb B.1
360	hMpxV/USA/WA-UW-0131/2022	EPI_ISL_14699946	North America / USA / Washington	Human	1	197176	33.00	Not Available	IIb B.1
361	hMpxV/USA/WA-UW-0135/2022	EPI_ISL_14699949	North America / USA / Washington	Human	1	197176	33.00	Not Available	IIb B.1

362	hMpxV/USA/WA-UW-0137/2022	EPI_ISL_14699950	North America / USA / Washington	Human	1	197178	33.00	Not Available	IIb B.1.1
363	hMpxV/USA/WA-UW-0145/2022	EPI_ISL_14699953	North America / USA / Washington	Human	1	197176	33.00	Not Available	IIb B.1
364	hMpxV/USA/WA-UW-0150/2022	EPI_ISL_14699955	North America / USA / Washington	Human	1	197176	33.00	Not Available	IIb B.1
365	hMpxV/USA/WA-UW-0067/2022	EPI_ISL_14699957	North America / USA / Washington	Human	1	197176	33.00	Not Available	IIb B.1
366	hMpxV/USA/WA-UW-0096/2022	EPI_ISL_14699958	North America / USA / Washington	Human	1	197176	33.00	Not Available	IIb B.1
367	hMpxV/USA/NE-UNMC-99/2022	EPI_ISL_14752091	North America / USA / Nebraska	Human	1	197182	33.00	Not Available	IIb B.1.11
368	hMpxV/USA/NE-UNMC-115/2022	EPI_ISL_14752094	North America / USA / Nebraska	Human	1	197180	33.00	Not Available	IIb B.1.11
369	hMpxV/USA/NE-UNMC-152/2022	EPI_ISL_14752096	North America / USA / Nebraska	Human	1	197174	33.00	Not Available	IIb B.1.3

370	hMpxV/USA/WA-UW-0160/2022	EPI_ISL_147 52260	North America / USA / Washington	Human	1	197190	33.00	Not Available	IIb B.1
371	hMpxV/USA/WA-UW-0171/2022	EPI_ISL_147 52263	North America / USA / Washington	Human	1	197194	33.00	Not Available	IIb B.1
372	hMpxV/Germany/un-RKI-289/2022	EPI_ISL_147 52264	Europe / Germany	Human	1	197139	33.01	Not Available	IIb B.1.1
373	hMpxV/Germany/un-RKI-290/2022	EPI_ISL_147 52265	Europe / Germany	Human	1	197139	33.01	Not Available	IIb B.1
374	hMpxV/Germany/un-RKI-294/2022	EPI_ISL_147 52272	Europe / Germany	Human	1	197139	33.01	Not Available	IIb B.1
375	hMpxV/Germany/un-RKI-295/2022	EPI_ISL_147 52274	Europe / Germany	Human	1	197139	33.01	Not Available	IIb B.1
376	hMpxV/United Kingdom/UKHSA-9000155/2022	EPI_ISL_147 52286	Europe / United Kingdom	Human	1	197212	33.01	Not Available	IIb A.2.1
377	hMpxV/United Kingdom/UKHSA-9000166/2022	EPI_ISL_147 52288	Europe / United Kingdom	Human	1	197196	33.01	Not Available	IIb A.2.2
378	hMpxV/USA/OK-UN-21/2022	EPI_ISL_148 04638	North America / USA / Oklahoma	Human	1	197178	33.00	Not Available	IIb B.1

379	hMpxV/USA/OK-UN-41/2022	EPI_ISL_14804639	North America / USA / Oklahoma	Human	1	197177	33.00	Not Available	IIb B.1
380	hMpxV/USA/OK-UN-67/2022	EPI_ISL_14804642	North America / USA / Oklahoma	Human	1	197175	33.00	Not Available	IIb B.1
381	hMpxV/USA/OK-UN-68/2022	EPI_ISL_14804643	North America / USA / Oklahoma	Human	1	197175	33.00	Not Available	IIb B.1.8
382	hMpxV/USA/OK-UN-69/2022	EPI_ISL_14804644	North America / USA / Oklahoma	Human	1	197176	33.00	Not Available	IIb B.1.5
383	hMpxV/USA/OK-UN-134/2022	EPI_ISL_14804646	North America / USA / Oklahoma	Human	1	197176	33.00	Not Available	IIb B.1.4
384	hMpxV/USA/OK-UN-149/2022	EPI_ISL_14804647	North America / USA / Oklahoma	Human	1	197174	33.00	Not Available	IIb B.1
385	hMpxV/Brazil/SP-IAL-63/2022	EPI_ISL_14809096	South America / Brazil / Sao Paulo / Sao Paulo	Human	1	196825	33.01	Lesion swab	IIb B.1.1
386	hMpxV/Peru/HUC-INS-121/2022	EPI_ISL_14818783	South America / Peru / Huanuco	Human	1	197193	33.00	Lesion swab	IIb B.1.6

387	hMpxV/Peru/LIM-INS-130/2022	EPI_ISL_14818792	South America / Peru / Lima	Human	1	197193	33.00	Lesion swab	IIb B.1.6
388	hMpxV/Peru/ARE-INS-139/2022	EPI_ISL_14818801	South America / Peru / Arequipa	Human	1	197193	33.00	Lesion swab	IIb B.1
389	hMpxV/Peru/LAM-INS-143/2022	EPI_ISL_14818805	South America / Peru / Lambayeque	Human	1	197194	33.00	Lesion swab	IIb B.1
390	hMpxV/Peru/LIM-INS-151/2022	EPI_ISL_14818813	South America / Peru / Lima	Human	1	197196	33.00	Lesion swab	IIb B.1.6
391	hMpxV/DRC/KWI-0158/2022	EPI_ISL_14838587	Africa / Democratic Republic of the Congo	Human	1	196851	33.08	Vesicles	I (probable IIb A)
392	hMpxV/France/un-IHU-00002/2022	EPI_ISL_14863050	Europe / France	Human	1	197172	33.00	Not Available	IIb B.1.1
393	hMpxV/France/un-IHU-00004/2022	EPI_ISL_14863052	Europe / France	Human	1	197155	33.00	Not Available	IIb B.1
394	hMpxV/Brazil/SP-IAL-68/2022	EPI_ISL_14865785	South America / Brazil / Sao Paulo / Sao Paulo	Human	1	197205	33.00	Lesion swab	IIb B.1.1
395	hMpxV/Brazil/SP-IAL-72/2022	EPI_ISL_14866752	South America / Brazil / Sao	Human	1	197205	33.00	Lesion swab	IIb B.1.1

			Paulo / Sao carlos						
396	hMpxV/USA/OH-UW-071356/2022	EPI_ISL_149 10863	North America / USA / Ohio	Human	1	197186	33.00	Not Available	IIb B.1
397	hMpxV/United Kingdom/UKHSA-9000220_NCPV_1703/2022	EPI_ISL_149 10886	Europe / United Kingdom	Human	1	197205	33.00	Not Available	IIb B.1
398	hMpxV/United Kingdom/UKHSA-9000289/2022	EPI_ISL_149 23901	Europe / United Kingdom	Human	1	197202	33.00	Buttock swab	IIb B.1.7
399	hMpxV/United Kingdom/UKHSA-9000353/2022	EPI_ISL_149 23904	Europe / United Kingdom	Human	1	197205	33.00	Not Available	IIb B.1
400	hMpxV/Austria/MUW-1533948/2022	EPI_ISL_149 34116	Europe / Austria / Graz	Human	1	197204	33.00	Lesion swab	IIb B.1
401	hMpxV/Austria/MUW-1538395/2022	EPI_ISL_149 34140	Europe / Austria / Lower Austria	Human	1	197205	33.00	Lesion swab	IIb B.1
402	hMpxV/Austria/MUW-1539124/2022	EPI_ISL_149 34382	Europe / Austria / Vienna	Human	1	197204	33.00	Lesion swab	IIb B.1
403	hMpxV/Austria/MUW-1540386/2022	EPI_ISL_149 34478	Europe / Austria / Vienna	Human	1	197204	33.00	Lesion swab	IIb B.1

404	hMpxV/United Kingdom/UKHSA-Qjmiyl_9000330/2022	EPI_ISL_149 34480	Europe / United Kingdom	Human	1	197207	33.00	Not Available	IIb B.1
405	hMpxV/United Kingdom/UKHSA-i0Eo3X_9000331/2022	EPI_ISL_149 34481	Europe / United Kingdom	Human	1	197205	33.00	Not Available	IIb B.1
406	hMpxV/United Kingdom/UKHSA-Mfh7qh_9000333/2022	EPI_ISL_149 34483	Europe / United Kingdom	Human	1	197205	33.00	Not Available	IIb B.1
407	hMpxV/United Kingdom/UKHSA-L0aaxP_9000335/2022	EPI_ISL_149 34485	Europe / United Kingdom	Human	1	197205	33.00	Not Available	IIb B.1.2
408	hMpxV/United Kingdom/UKHSA-i96eY5_9000346/2022	EPI_ISL_149 34486	Europe / United Kingdom	Human	1	197205	33.00	face, chest, arms swab	IIb B.1
409	hMpxV/United Kingdom/UKHSA-3LjZEY_9000355/2022	EPI_ISL_149 34492	Europe / United Kingdom	Human	1	197205	33.00	rectal swab	IIb B.1
410	hMpxV/United Kingdom/UKHSA-yttrq1_9000358/2022	EPI_ISL_149 34494	Europe / United Kingdom	Human	1	197205	33.00	lesion swab	IIb B.1
411	hMpxV/United Kingdom/UKHSA-QfgFmq_9000359/2022	EPI_ISL_149 34495	Europe / United Kingdom	Human	1	197207	33.00	lesion swab	IIb B.1

412	hMpxV/United Kingdom/UKHSA-Yob5K4_9000201/2022	EPI_ISL_14934622	Europe / United Kingdom	Human	1	197205	33.00	Not Available	IIb B.1.7
413	hMpxV/United Kingdom/UKHSA-Yob5K4_9000202/2022	EPI_ISL_14934623	Europe / United Kingdom	Human	1	197205	33.00	Not Available	IIb B.1.7
414	hMpxV/United Kingdom/UKHSA-cmjxZM_9000205/2022	EPI_ISL_14934626	Europe / United Kingdom	Human	1	197205	33.00	Not Available	IIb B.1.7
415	hMpxV/United Kingdom/UKHSA-2v76E1_9000221/2022	EPI_ISL_14934637	Europe / United Kingdom	Human	1	197205	33.00	Not Available	IIb B.1.7
416	hMpxV/United Kingdom/UKHSA-2v76E1_9000222/2022	EPI_ISL_14934638	Europe / United Kingdom	Human	1	197205	33.00	Not Available	IIb B.1.7
417	hMpxV/United Kingdom/UKHSA-K1dQNt_9000224/2022	EPI_ISL_14934640	Europe / United Kingdom	Human	1	197205	33.00	Not Available	IIb B.1.7
418	hMpxV/United Kingdom/UKHSA-K1dQNt_9000225/2022	EPI_ISL_14934641	Europe / United Kingdom	Human	1	197205	33.00	Not Available	IIb B.1.7
419	hMpxV/United Kingdom/UKHSA-fZLVcc_9000227/2022	EPI_ISL_14934642	Europe / United Kingdom	Human	1	197205	33.00	Not Available	IIb B.1.2

420	hMpxV/United Kingdom/UKHSA-TcUINH_9000230/2022	EPI_ISL_14934645	Europe / United Kingdom	Human	1	197205	33.00	Not Available	IIb B.1.2
421	hMpxV/United Kingdom/UKHSA-BhVKjm_9000233/2022	EPI_ISL_14934647	Europe / United Kingdom	Human	1	197208	33.00	Not Available	IIb B.1.12
422	hMpxV/United Kingdom/UKHSA-Fap1fz_9000235/2022	EPI_ISL_14934649	Europe / United Kingdom	Human	1	197205	33.00	Not Available	IIb B.1.2
423	hMpxV/United Kingdom/UKHSA-KMgssq_9000236/2022	EPI_ISL_14934650	Europe / United Kingdom	Human	1	197205	33.00	Not Available	IIb B.1
424	hMpxV/United Kingdom/UKHSA-UbfBKN_9000238/2022	EPI_ISL_14934652	Europe / United Kingdom	Human	1	197205	33.00	Not Available	IIb B.1
425	hMpxV/United Kingdom/UKHSA-42KDT6_9000240/2022	EPI_ISL_14934654	Europe / United Kingdom	Human	1	197205	33.00	Not Available	IIb B.1.7
426	hMpxV/United Kingdom/UKHSA-42KDT6_9000241/2022	EPI_ISL_14934655	Europe / United Kingdom	Human	1	197205	33.00	Not Available	IIb B.1.7
427	hMpxV/United Kingdom/UKHSA-OXSHAI_9000245/2022	EPI_ISL_14934656	Europe / United Kingdom	Human	1	197205	33.00	Not Available	IIb B.1

428	hMpxV/United Kingdom/UKHSA-V5dRAI_9000247/2022	EPI_ISL_149 34658	Europe / United Kingdom	Human	1	197205	33.00	Not Available	IIb B.1.7
429	hMpxV/United Kingdom/UKHSA-AooXJI_9000248/2022	EPI_ISL_149 34659	Europe / United Kingdom	Human	1	197205	33.00	Not Available	IIb B.1.7
430	hMpxV/United Kingdom/UKHSA-jpMpSR_9000252/2022	EPI_ISL_149 34663	Europe / United Kingdom	Human	1	197205	33.00	Not Available	IIb B.1
431	hMpxV/United Kingdom/UKHSA-k9tr7U_9000256/2022	EPI_ISL_149 34666	Europe / United Kingdom	Human	1	197202	33.00	Not Available	IIb B.1
432	hMpxV/United Kingdom/UKHSA-UBm6ar_9000259/2022	EPI_ISL_149 34668	Europe / United Kingdom	Human	1	197205	33.00	Not Available	IIb B.1.2
433	hMpxV/United Kingdom/UKHSA-8gbW2X_9000262/2022	EPI_ISL_149 34671	Europe / United Kingdom	Human	1	197179	33.00	Not Available	IIb B.1
434	hMpxV/United Kingdom/UKHSA-qQzToC_9000264/2022	EPI_ISL_149 34673	Europe / United Kingdom	Human	1	197205	33.00	Not Available	IIb B.1
435	hMpxV/United Kingdom/UKHSA-GGr0lD_9000276/2022	EPI_ISL_149 34678	Europe / United Kingdom	Human	1	197207	33.00	Not Available	IIb B.1.7

436	hMpxV/United Kingdom/UKHSA-cAC1lu_9000277/2022	EPI_ISL_14934679	Europe / United Kingdom	Human	1	197205	33.00	Not Available	IIb B.1
437	hMpxV/United Kingdom/UKHSA-vSkiJH_9000281/2022	EPI_ISL_14934683	Europe / United Kingdom	Human	1	197205	33.00	Not Available	IIb B.1.7
438	hMpxV/United Kingdom/UKHSA-z0MIuQ_9000282/2022	EPI_ISL_14934684	Europe / United Kingdom	Human	1	197203	33.00	Not Available	IIb B.1.3
439	hMpxV/United Kingdom/UKHSA-jW1uT9_9000283/2022	EPI_ISL_14934685	Europe / United Kingdom	Human	1	197207	33.00	Not Available	IIb B.1
440	hMpxV/United Kingdom/UKHSA-XJRG9b_9000293/2022	EPI_ISL_14934688	Europe / United Kingdom	Human	1	197179	33.00	Not Available	IIb B.1
441	hMpxV/United Kingdom/UKHSA-eobXCZ_9000296/2022	EPI_ISL_14934691	Europe / United Kingdom	Human	1	197205	33.00	Not Available	IIb B.1.2
442	hMpxV/United Kingdom/UKHSA-WOcPOH_9000297/2022	EPI_ISL_14934692	Europe / United Kingdom	Human	1	197205	33.00	Not Available	IIb B.1.7
443	hMpxV/United Kingdom/UKHSA-cbQgLw_9000299/2022	EPI_ISL_14934693	Europe / United Kingdom	Human	1	197205	33.00	Not Available	IIb B.1.2

444	hMpxV/United Kingdom/UKHSA-imBNaw_9000300/2022	EPI_ISL_149 34694	Europe / United Kingdom	Human	1	197205	33.00	Not Available	IIb B.1.8
445	hMpxV/United Kingdom/UKHSA-jTHLhj_9000307/2022	EPI_ISL_149 34696	Europe / United Kingdom	Human	1	197205	33.00	Not Available	IIb B.1
446	hMpxV/United Kingdom/UKHSA-eunRrC_9000312/2022	EPI_ISL_149 34697	Europe / United Kingdom	Human	1	197205	33.00	Not Available	IIb B.1
447	hMpxV/United Kingdom/UKHSA-VghSe2_9000318/2022	EPI_ISL_149 34699	Europe / United Kingdom	Human	1	197205	33.00	Not Available	IIb B.1
448	hMpxV/United Kingdom/UKHSA-PZ8OdF_9000319/2022	EPI_ISL_149 34700	Europe / United Kingdom	Human	1	197205	33.00	Not Available	IIb B.1
449	hMpxV/United Kingdom/UKHSA-1bN2W6_9000324/2022	EPI_ISL_149 34703	Europe / United Kingdom	Human	1	197205	33.00	Not Available	IIb B.1
450	hMpxV/United Kingdom/UKHSA-7eECz2_9000326/2022	EPI_ISL_149 34705	Europe / United Kingdom	Human	1	197205	33.00	Not Available	IIb B.1.2
451	hMpxV/United Kingdom/UKHSA-rYdyAi_9000329/2022	EPI_ISL_149 34707	Europe / United Kingdom	Human	1	197205	33.00	Not Available	IIb B.1

452	hMpxV/Hong Kong/HKU-220914-001/2022	EPI_ISL_14945299	Asia / Hong Kong	Human	1	197426	32.97	Vesicle fluid	IIb B.1.7
453	hMpxV/USA/NE-UNMC-199/2022	EPI_ISL_14977310	North America / USA / Nebraska	Human	1	197191	33.00	Not Available	IIb B.1.3
454	hMpxV/Brazil/SP-IAL-75/2022	EPI_ISL_14995578	South America / Brazil / Sao Paulo / Ilhabela	Human	1	196820	33.01	Lesion swab	IIb B.1.1
455	hMpxV/Brazil/SP-IAL-77/2022	EPI_ISL_14995580	South America / Brazil / Sao Paulo / Ribeirao Preto	Human	1	196491	33.01	Lesion swab	IIb B.1.1
456	hMpxV/Brazil/SP-IAL-83/2022	EPI_ISL_14995587	South America / Brazil / Sao Paulo / Itaquaquecetuba	Human	1	197205	33.00	Lesion swab	IIb B.1
457	hMpxV/USA/OH-UW-070197/2022	EPI_ISL_14997063	North America / USA / Ohio	Human	1	197175	33.00	Not Available	IIb B.1
458	hMpxV/USA/OH-UW-070832/2022	EPI_ISL_14997064	North America / USA / Ohio	Human	1	197170	33.00	Not Available	IIb B.1.8
459	hMpxV/USA/OH-UW-071048/2022	EPI_ISL_14997065	North America / USA / Ohio	Human	1	197177	33.00	Not Available	IIb B.1

460	hMpxV/USA/WA-UW-082488/2022	EPI_ISL_14997068	North America / USA / Washington	Human	1	197178	33.00	Not Available	IIb B.1.8
461	hMpxV/USA/WA-UW-084331/2022	EPI_ISL_14997069	North America / USA / Washington	Human	1	197178	33.00	Not Available	IIb B.1.4
462	hMpxV/Germany/un-RKI-340/2022	EPI_ISL_15076132	Europe / Germany	Human	1	197139	33.01	Swab	IIb B.1.1
463	hMpxV/Germany/un-RKI-347/2022	EPI_ISL_15076137	Europe / Germany	Human	1	197139	33.01	Swab	IIb B.1.2
464	hMpxV/Germany/un-RKI-350/2022	EPI_ISL_15076140	Europe / Germany	Human	1	197139	33.01	Swab	IIb B.1.1
465	hMpxV/Germany/un-RKI-351/2022	EPI_ISL_15076141	Europe / Germany	Human	1	197140	33.01	Swab	IIb B.1.1
466	hMpxV/Germany/un-RKI-352/2022	EPI_ISL_15076142	Europe / Germany	Human	1	196226	33.01	Swab	IIb B.1
467	hMpxV/Germany/un-RKI-353/2022	EPI_ISL_15076143	Europe / Germany	Human	1	197139	33.01	Swab	IIb B.1
468	hMpxV/Germany/un-RKI-354/2022	EPI_ISL_15076144	Europe / Germany	Human	1	197139	33.01	Swab	IIb B.1
469	hMpxV/Germany/un-RKI-357/2022	EPI_ISL_15076147	Europe / Germany	Human	1	197140	33.01	Swab	IIb B.1.7

470	hMpxV/Germany/un-RKI-360/2022	EPI_ISL_15076150	Europe / Germany	Human	1	197139	33.01	Swab	IIb B.1
471	hMpxV/Germany/un-RKI-361/2022	EPI_ISL_15076151	Europe / Germany	Human	1	197139	33.01	Swab	IIb B.1.7
472	hMpxV/Germany/un-RKI-362/2022	EPI_ISL_15076152	Europe / Germany	Human	1	197140	33.01	Swab	IIb B.1.10
473	hMpxV/USA/NC-UNC-0003/2022	EPI_ISL_15076180	North America / USA / North Carolina	Human	1	197200	33.00	Lesion swab	IIb B.1.7
474	hMpxV/Germany/un-RKI-379/2022	EPI_ISL_15116268	Europe / Germany	Human	1	197140	33.01	swab	IIb B.1
475	hMpxV/Germany/un-RKI-382/2022	EPI_ISL_15116271	Europe / Germany	Human	1	196227	33.01	swab	IIb B.1
476	hMpxV/Germany/un-RKI-384/2022	EPI_ISL_15116273	Europe / Germany	Human	1	197140	33.01	swab	IIb B.1
477	hMpxV/Germany/un-RKI-385/2022	EPI_ISL_15116274	Europe / Germany	Human	1	197140	33.01	swab	IIb B.1
478	hMpxV/Germany/un-RKI-388/2022	EPI_ISL_15116277	Europe / Germany	Human	1	197139	33.00	swab	IIb B.1.1
479	hMpxV/Germany/un-RKI-390/2022	EPI_ISL_15116279	Europe / Germany	Human	1	197139	33.01	swab	IIb B.1
480	hMpxV/Germany/un-RKI-392/2022	EPI_ISL_15116281	Europe / Germany	Human	1	197140	33.01	swab	IIb B.1.3

481	hMpxV/Germany/un-RKI-402/2022	EPI_ISL_15116290	Europe / Germany	Human	1	197139	33.01	swab	IIb B.1.1
482	hMpxV/Germany/un-RKI-403/2022	EPI_ISL_15116291	Europe / Germany	Human	1	197139	33.01	swab	IIb B.1.1
483	hMpxV/Germany/un-RKI-406/2022	EPI_ISL_15116295	Europe / Germany	Human	1	197140	33.01	swab	IIb B.1.5
484	hMpxV/Germany/un-RKI-408/2022	EPI_ISL_15116297	Europe / Germany	Human	1	197139	33.01	swab	IIb B.1
485	hMpxV/Germany/un-RKI-409/2022	EPI_ISL_15116298	Europe / Germany	Human	1	197140	33.01	swab	IIb B.1
486	hMpxV/Germany/un-RKI-410/2022	EPI_ISL_15116299	Europe / Germany	Human	1	197139	33.01	swab	IIb B.1.7
487	hMpxV/USA/MN-CDC-0001/2022	EPI_ISL_15116301	North America / USA / Minnesota	Human	1	205032	32.73	lesion swab	IIb B.1.3
488	hMpxV/USA/FL-CDC-0065/2022	EPI_ISL_15116303	North America / USA / Florida	Human	1	200014	32.81	lesion swab	IIb B.1.13
489	hMpxV/USA/RI-CDC-0001/2022	EPI_ISL_15116305	North America / USA / Rhode Island	Human	1	206803	32.99	lesion swab	IIb B.1
490	hMpxV/Gabon/BNITM-0001/1988	EPI_ISL_15158314	Africa / Gabon	Human	1	196640	33.09	Not Available	I (probable IIb A)

491	hMpxV/United Kingdom/UKHSA-9000365/2022	EPI_ISL_15158400	Europe / United Kingdom	Human	1	197194	33.00	Vesicles swab	IIb B.1
492	hMpxV/United Kingdom/UKHSA-9000366/2022	EPI_ISL_15158401	Europe / United Kingdom	Human	1	197192	33.00	Swab	IIb B.1.3
493	hMpxV/United Kingdom/UKHSA-9000368/2022	EPI_ISL_15158402	Europe / United Kingdom	Human	1	197196	33.00	Vesicular fluid swab	IIb B.1
494	hMpxV/United Kingdom/UKHSA-9000369/2022	EPI_ISL_15158403	Europe / United Kingdom	Human	1	197194	33.00	Lesion swab left arm	IIb B.1.2
495	hMpxV/United Kingdom/UKHSA-9000370/2022	EPI_ISL_15158404	Europe / United Kingdom	Human	1	197192	33.00	Penile swab	IIb B.1.3
496	hMpxV/United Kingdom/UKHSA-9000374/2022	EPI_ISL_15158407	Europe / United Kingdom	Human	1	197194	33.00	Swab	IIb B.1
497	hMpxV/United Kingdom/UKHSA-9000375/2022	EPI_ISL_15158408	Europe / United Kingdom	Human	1	197194	33.00	Lesion swab left arm	IIb B.1.7
498	hMpxV/United Kingdom/UKHSA-9000378/2022	EPI_ISL_15158411	Europe / United Kingdom	Human	1	197194	33.00	Skin swab	IIb B.1

499	hMpxV/United Kingdom/UKHSA-9000380/2022	EPI_ISL_15158412	Europe / United Kingdom	Human	1	197196	33.00	Swab	IIb B.1
500	hMpxV/United Kingdom/UKHSA-9000384/2022	EPI_ISL_15158416	Europe / United Kingdom	Human	1	197194	33.00	Swab	IIb B.1.2
501	hMpxV/United Kingdom/UKHSA-9000387/2022	EPI_ISL_15158418	Europe / United Kingdom	Human	1	197194	33.00	Perianal lesion swab	IIb B.1.6
502	hMpxV/United Kingdom/UKHSA-9000390/2022	EPI_ISL_15158421	Europe / United Kingdom	Human	1	197194	33.00	Lesion swab trunk	IIb B.1.2
503	hMpxV/United Kingdom/UKHSA-9000392/2022	EPI_ISL_15158423	Europe / United Kingdom	Human	1	197198	33.00	Lesion swab chest	IIb B.1
504	hMpxV/United Kingdom/UKHSA-9000393/2022	EPI_ISL_15158424	Europe / United Kingdom	Human	1	197196	33.00	Vesicle swab	IIb B.1
505	hMpxV/United Kingdom/UKHSA-9000395/2022	EPI_ISL_15158426	Europe / United Kingdom	Human	1	197198	33.00	Lesion swab	IIb B.1.2
506	hMpxV/United Kingdom/UKHSA-9000396/2022	EPI_ISL_15158427	Europe / United Kingdom	Human	1	197196	33.00	Lesion swab	IIb B.1.7

507	hMpxV/United Kingdom/UKHSA-9000397/2022	EPI_ISL_15158428	Europe / United Kingdom	Human	1	197196	33.00	Not Available	IIb B.1.7
508	hMpxV/United Kingdom/UKHSA-9000399/2022	EPI_ISL_15158430	Europe / United Kingdom	Human	1	197194	33.00	Swab	IIb B.1.7
509	hMpxV/United Kingdom/UKHSA-9000403/2022	EPI_ISL_15158432	Europe / United Kingdom	Human	1	197191	33.00	Swab	IIb B.1.3
510	hMpxV/United Kingdom/UKHSA-9000411/2022	EPI_ISL_15158439	Europe / United Kingdom	Human	1	197194	33.00	Not Available	IIb B.1
511	hMpxV/United Kingdom/UKHSA-9000413/2022	EPI_ISL_15158441	Europe / United Kingdom	Human	1	197196	33.00	Arm swab	IIb B.1
512	hMpxV/United Kingdom/UKHSA-9000415/2022	EPI_ISL_15158442	Europe / United Kingdom	Human	1	197196	33.00	Not Available	IIb B.1.7
513	hMpxV/United Kingdom/UKHSA-9000419/2022	EPI_ISL_15158446	Europe / United Kingdom	Human	1	197196	33.00	Vesicle swab	IIb B.1
514	hMpxV/United Kingdom/UKHSA-9000421/2022	EPI_ISL_15158448	Europe / United Kingdom	Human	1	197194	33.00	Not Available	IIb B.1.3

515	hMpxV/United Kingdom/UKHSA-9000422/2022	EPI_ISL_15158449	Europe / United Kingdom	Human	1	197192	33.00	Not Available	IIb B.1.7
516	hMpxV/United Kingdom/UKHSA-9000438/2022	EPI_ISL_15158460	Europe / United Kingdom	Human	1	197194	33.00	Hand swab	IIb B.1.2
517	hMpxV/United Kingdom/UKHSA-9000442/2022	EPI_ISL_15158461	Europe / United Kingdom	Human	1	197193	33.00	Groin lesion swab	IIb B.1
518	hMpxV/United Kingdom/UKHSA-9000451/2022	EPI_ISL_15158466	Europe / United Kingdom	Human	1	197198	33.00	Lesion swab	IIb B.1
519	hMpxV/Italy/SIC-AOUP-UNIPA_FD/2022	EPI_ISL_15263355	Europe / Italy / Sicily	Human	1	196913	33.03	Skin lesion	IIb B.1
520	hMpxV/Netherlands/un-EMC-NL055/2022	EPI_ISL_15269699	Europe / Netherlands	Human	1	197109	33.01	Not Available	IIb B.1
521	hMpxV/Germany/un-RKI432/2022	EPI_ISL_15283601	Europe / Germany	Human	1	197140	33.01	Swab	IIb B.1
522	hMpxV/Germany/un-RKI433/2022	EPI_ISL_15283603	Europe / Germany	Human	1	197139	33.01	Swab	IIb B.1
523	hMpxV/Egypt/MOH-NRC-0002/2022	EPI_ISL_15292947	Africa / Egypt	Human	1	197201	33.01	Not Available	IIb A

524	hMpxV/Spain/un-IHC-2428/2022	EPI_ISL_15292976	Europe / Spain	Human	1	197933	32.93	Not Available	IIb B.1.1
525	hMpxV/Spain/un-IHC-419/2022	EPI_ISL_15292979	Europe / Spain	Human	1	197833	32.95	Not Available	IIb B.1
526	hMpxV/Spain/un-IHC-415/2022	EPI_ISL_15292982	Europe / Spain	Human	1	197939	32.94	Not Available	IIb B.1
527	hMpxV/Spain/un-IHC-417/2022	EPI_ISL_15292983	Europe / Spain	Human	1	197839	32.95	Not Available	IIb B.1.10
528	hMpxV/Spain/un-IHC-347_R/2022	EPI_ISL_15292984	Europe / Spain	Human	1	197918	32.94	Not Available	IIb B.1.8
529	hMpxV/Spain/un-IHC-431/2022	EPI_ISL_15292986	Europe / Spain	Human	1	197833	32.95	Not Available	IIb B.1
530	hMpxV/Spain/un-IHC-2317/2022	EPI_ISL_15292989	Europe / Spain	Human	1	197923	32.94	Not Available	IIb B.1
531	hMpxV/Spain/un-IHC-2318/2022	EPI_ISL_15292995	Europe / Spain	Human	1	197838	32.95	Not Available	IIb B.1
532	hMpxV/Spain/un-IHC-438/2022	EPI_ISL_15292997	Europe / Spain	Human	1	197838	32.95	Not Available	IIb B.1
533	hMpxV/Spain/un-IHC-1075/2022	EPI_ISL_15292998	Europe / Spain	Human	1	197865	32.94	Not Available	IIb B.1
534	hMpxV/Spain/un-IHC-420/2022	EPI_ISL_15292999	Europe / Spain	Human	1	197907	32.94	Not Available	IIb B.1

535	hMpxV/Spain/un-IHC-698/2022	EPI_ISL_15293002	Europe / Spain	Human	1	197967	32.93	Not Available	IIb B.1.1
536	hMpxV/Spain/un-IHC-411/2022	EPI_ISL_15293006	Europe / Spain	Human	1	197895	32.94	Not Available	IIb B.1.7
537	hMpxV/Spain/un-IHC-416/2022	EPI_ISL_15293007	Europe / Spain	Human	1	197930	32.94	Not Available	IIb B.1
538	hMpxV/Spain/un-IHC-900/2022	EPI_ISL_15293008	Europe / Spain	Human	1	197907	32.94	Not Available	IIb B.1
539	hMpxV/Spain/un-IHC-418/2022	EPI_ISL_15293009	Europe / Spain	Human	1	197919	32.94	Not Available	IIb B.1
540	hMpxV/USA/NE-UNMC-211/2022	EPI_ISL_15332336	North America / USA / Nebraska	Human	1	197188	33.00	Not Available	IIb B.1.3
541	hMpxV/USA/NE-UNMC-230/2022	EPI_ISL_15332337	North America / USA / Nebraska	Human	1	197205	33.00	Not Available	IIb B.1.3
542	hMpxV/USA/OK-UNMC-288/2022	EPI_ISL_15332338	North America / USA / Oklahoma	Human	1	197196	33.00	Not Available	IIb B.1.7
543	hMpxV/USA/OK-UNMC-301/2022	EPI_ISL_15332339	North America / USA / Oklahoma	Human	1	197199	33.00	Not Available	IIb B.1.2

544	hMpxV/USA/WA-UW-080941/2022	EPI_ISL_15367959	North America / USA / Washington	Human	1	197192	33.00	Not Available	IIb B.1
545	hMpxV/USA/WA-UW-081326/2022	EPI_ISL_15367961	North America / USA / Washington	Human	1	197196	33.00	Not Available	IIb B.1.11
546	hMpxV/USA/WA-UW-081603/2022	EPI_ISL_15367964	North America / USA / Washington	Human	1	197194	33.00	Not Available	IIb B.1
547	hMpxV/USA/WA-UW-082483/2022	EPI_ISL_15367966	North America / USA / Washington	Human	1	197190	33.00	Not Available	IIb B.1
548	hMpxV/USA/WA-UW-084263/2022	EPI_ISL_15367977	North America / USA / Washington	Human	1	197193	33.00	Not Available	IIb B.1.11
549	hMpxV/USA/WA-UW-084573/2022	EPI_ISL_15367979	North America / USA / Washington	Human	1	197192	33.00	Not Available	IIb B.1
550	hMpxV/USA/WA-UW-085850/2022	EPI_ISL_15367986	North America / USA / Washington	Human	1	197192	33.00	Not Available	IIb B.1.3
551	hMpxV/USA/WA-UW-086901/2022	EPI_ISL_15367991	North America / USA / Washington	Human	1	197193	33.00	Not Available	IIb B.1.11

552	hMpxV/USA/WA-UW-088353/2022	EPI_ISL_15368004	North America / USA / Washington	Human	1	197195	33.00	Not Available	IIb B.1
553	hMpxV/USA/WA-UW-089475/2022	EPI_ISL_15368005	North America / USA / Washington	Human	1	197192	33.00	Not Available	IIb B.1
554	hMpxV/USA/WA-UW-098055/2022	EPI_ISL_15368011	North America / USA / Washington	Human	1	197190	33.00	Not Available	IIb B.1
555	hMpxV/Nigeria/CBR-040/2019	EPI_ISL_15370073	Africa / Nigeria	Human	1	197052	33.02	pustule swabs	IIb A.1
556	hMpxV/Nigeria/CBR-046/2019	EPI_ISL_15370075	Africa / Nigeria	Human	1	197514	32.98	pustule swabs	IIb A
557	hMpxV/Nigeria/CBR-053/2019	EPI_ISL_15370077	Africa / Nigeria	Human	1	197135	33.01	pustule swabs	IIb A.1
558	hMpxV/Philippines/RITM-002/2022	EPI_ISL_15380492	Asia / Philippines / National Capital Region	Human	1	197119	33.01	Lesion base dry swab lesion roof	IIb B.1
559	hMpxV/USA/OH-UW-079847/2022	EPI_ISL_15384399	North America / USA / Ohio	Human	1	197176	33.00	Not Available	IIb B.1.13
560	hMpxV/USA/OH-UW-076485/2022	EPI_ISL_15384401	North America / USA / Ohio	Human	1	197168	33.00	Not Available	IIb B.1

561	hMpxV/USA/WA-UW-074224/2022	EPI_ISL_15384402	North America / USA / Washington	Human	1	197176	33.00	Not Available	IIb B.1
562	hMpxV/USA/WA-UW-074184/2022	EPI_ISL_15384403	North America / USA / Washington	Human	1	197178	33.00	Not Available	IIb B.1.1
563	hMpxV/USA/OH-UW-072752/2022	EPI_ISL_15384404	North America / USA / Ohio	Human	1	197176	33.00	Not Available	IIb B.1.2
564	hMpxV/USA/WA-UW-071966/2022	EPI_ISL_15384406	North America / USA / Washington	Human	1	197174	33.00	Not Available	IIb B.1.1
565	hMpxV/USA/WA-UW-075687/2022	EPI_ISL_15384407	North America / USA / Washington	Human	1	197174	33.00	Not Available	IIb B.1.3
566	hMpxV/Germany/un-RKI328/2022	EPI_ISL_15390495	Europe / Germany	Human	1	197139	33.01	Swab	IIb B.1.1
567	hMpxV/Germany/un-RKI332/2022	EPI_ISL_15390499	Europe / Germany	Human	1	197139	33.01	Swab	IIb B.1
568	hMpxV/Germany/un-RKI333/2022	EPI_ISL_15390500	Europe / Germany	Human	1	197139	33.01	Swab	IIb B.1.5
569	hMpxV/Brazil/SP-IAL-102/2022	EPI_ISL_15419133	South America / Brazil / Sao Paulo /	Human	1	196805	33.01	Lesion swab	IIb B.1

			Presidente Prudente						
570	hMpxV/Brazil/SP-IAL-103/2022	EPI_ISL_154 19134	South America / Brazil / Sao Paulo / Caieiras	Human	1	196715	33.01	Lesion swab	IIb B.1
571	hMpxV/Brazil/SP-IAL-104/2022	EPI_ISL_154 19135	South America / Brazil / Sao Paulo / Sao Paulo	Human	1	196806	33.01	Lesion swab	IIb B.1
572	hMpxV/Brazil/SP-IAL-105/2022	EPI_ISL_154 19136	South America / Brazil / Sao Paulo / Sao Paulo	Human	1	196762	33.01	Lesion swab	IIb B.1
573	hMpxV/Brazil/SP-IAL-106/2022	EPI_ISL_154 19137	South America / Brazil / Sao Paulo / Jandira	Human	1	196751	33.01	Lesion swab	IIb B.1
574	hMpxV/Brazil/SP-IAL-107/2022	EPI_ISL_154 19138	South America / Brazil / Sao Paulo / Pirassununga	Human	1	196495	33.01	Lesion swab	IIb B.1
575	hMpxV/Brazil/SP-IAL-110/2022	EPI_ISL_154 19141	South America / Brazil / Sao Paulo / Sao Paulo	Human	1	196530	33.01	Lesion swab	IIb B.1

576	hMpxV/Brazil/SP-IAL-112/2022	EPI_ISL_15419143	South America / Brazil / Sao Paulo / Barretos	Human	1	196761	33.01	Lesion swab	IIb B.1
577	hMpxV/Brazil/SP-IAL-114/2022	EPI_ISL_15419145	South America / Brazil / Sao Paulo / Osasco	Human	1	196558	33.01	Lesion swab	IIb B.1
578	hMpxV/Brazil/SP-IAL-115/2022	EPI_ISL_15419146	South America / Brazil / Sao Paulo / Sao Paulo	Human	1	196909	33.00	Lesion swab	IIb B.1
579	hMpxV/Brazil/SP-IAL-116/2022	EPI_ISL_15419147	South America / Brazil / Sao Paulo / Diadema	Human	1	196610	33.01	Lesion swab	IIb B.1
580	hMpxV/Brazil/SP-IAL-122/2022	EPI_ISL_15419153	South America / Brazil / Sao Paulo / Sao Paulo	Human	1	196600	33.01	Lesion swab	IIb B.1
581	hMpxV/Brazil/SP-IAL-123/2022	EPI_ISL_15419154	South America / Brazil / Sao Paulo / Sao Jose do Rio Preto	Human	1	196475	33.01	Lesion swab	IIb B.1

582	hMpxV/Brazil/SP-IAL-124/2022	EPI_ISL_15419155	South America / Brazil / Sao Paulo / Atibaia	Human	1	196630	33.01	Lesion swab	IIb B.1
583	hMpxV/Brazil/SP-IAL-125/2022	EPI_ISL_15419156	South America / Brazil / Sao Paulo / Sao Paulo	Human	1	196785	33.01	Lesion swab	IIb B.1
584	hMpxV/Brazil/SP-IAL-130/2022	EPI_ISL_15419161	South America / Brazil / Sao Paulo / Jaboticabal	Human	1	196490	33.01	Lesion swab	IIb B.1
585	hMpxV/Brazil/SP-IAL-131/2022	EPI_ISL_15419162	South America / Brazil / Sao Paulo / Sao Paulo	Human	1	196493	33.01	Lesion swab	IIb B.1
586	hMpxV/USA/OK-UNMC-277/2022	EPI_ISL_15458903	North America / USA / Oklahoma	Human	1	197196	33.00	Not Available	IIb B.1.2
587	hMpxV/USA/OK-UNMC-273/2022	EPI_ISL_15458904	North America / USA / Oklahoma	Human	1	197199	33.00	Not Available	IIb B.1.2
588	hMpxV/USA/WA-UW-092912/2022	EPI_ISL_15608026	North America / USA / Washington	Human	1	197188	33.00	Not Available	IIb B.1

589	hMpxV/USA/WA-UW-093226/2022	EPI_ISL_15608028	North America / USA / Washington	Human	1	197193	33.00	Not Available	IIb B.1.11
590	hMpxV/USA/WA-UW-098348/2022	EPI_ISL_15608033	North America / USA / Washington	Human	1	197193	33.00	Not Available	IIb B.1.11
591	hMpxV/USA/un-UW-102378/2022	EPI_ISL_15608045	North America / USA	Human	1	197185	33.00	Not Available	IIb B.1
592	hMpxV/USA/WA-UW-108943/2022	EPI_ISL_15608046	North America / USA / Washington	Human	1	197194	33.00	Not Available	IIb B.1.2
593	hMpxV/USA/WA-UW-085359/2022	EPI_ISL_15608048	North America / USA / Washington	Human	1	197184	33.00	Not Available	IIb B.1.1
594	hMpxV/USA/WA-UW-096615/2022	EPI_ISL_15608049	North America / USA / Washington	Human	1	197196	33.00	Not Available	IIb B.1.11
595	hMpxV/USA/WA-UW-095970/2022	EPI_ISL_15608052	North America / USA / Washington	Human	1	197198	33.00	Not Available	IIb B.1.11
596	hMpxV/USA/WA-UW-109000/2022	EPI_ISL_15608055	North America / USA / Washington	Human	1	197205	33.00	Not Available	IIb B.1.11

597	hMpxV/USA/WA-UW-104793/2022	EPI_ISL_15608057	North America / USA / Washington	Human	1	197191	33.00	Not Available	IIb B.1
598	hMpxV/USA/un-UW-093679/2022	EPI_ISL_15608058	North America / USA	Human	1	197192	33.00	Not Available	IIb B.1.2
599	hMpxV/USA/NV-SNPHL-686071/2022	EPI_ISL_15608909	North America / USA / Nevada / Clark	Human	1	197176	33.00	Not Available	IIb B.1
600	hMpxV/Netherlands/NH-AUMC-0004/2022	EPI_ISL_15641543	Europe / Netherlands / North-Holland / Amsterdam	Human	1	196995	33.02	Swab sample	IIb B.1
601	hMpxV/Netherlands/NH-AUMC-0012/2022	EPI_ISL_15641550	Europe / Netherlands / North-Holland / Amsterdam	Human	1	196995	33.02	Swab sample	IIb B.1
602	hMpxV/Netherlands/NH-AUMC-0013/2022	EPI_ISL_15641551	Europe / Netherlands / North-Holland / Amsterdam	Human	1	196995	33.02	Swab sample	IIb B.1.14
603	hMpxV/Netherlands/NH-AUMC-0015/2022	EPI_ISL_15641553	Europe / Netherlands / North-Holland / Amsterdam	Human	1	196997	33.02	Swab sample	IIb B.1

604	hMpxV/Netherlands/NH-AUMC-0017/2022	EPI_ISL_156 41555	Europe / Netherlands / North-Holland / Amsterdam	Human	1	196995	33.02	Swab sample	IIb B.1.11
605	hMpxV/Netherlands/NH-AUMC-0022/2022	EPI_ISL_156 41560	Europe / Netherlands / North-Holland / Amsterdam	Human	1	196999	33.02	Swab sample	IIb B.1.2
606	hMpxV/Netherlands/NH-AUMC-0023/2022	EPI_ISL_156 41561	Europe / Netherlands / North-Holland / Amsterdam	Human	1	196511	33.01	Swab sample	IIb B.1.14
607	hMpxV/Netherlands/NH-AUMC-0024/2022	EPI_ISL_156 41562	Europe / Netherlands / North-Holland / Amsterdam	Human	1	196997	33.02	Swab sample	IIb B.1
608	hMpxV/Netherlands/NH-AUMC-0026/2022	EPI_ISL_156 41564	Europe / Netherlands / North-Holland / Amsterdam	Human	1	196999	33.02	Swab sample	IIb B.1
609	hMpxV/Netherlands/NH-AUMC-0028/2022	EPI_ISL_156 41566	Europe / Netherlands / North-Holland / Amsterdam	Human	1	196997	33.02	Swab sample	IIb B.1
610	hMpxV/Netherlands/NH-AUMC-0030/2022	EPI_ISL_156 41568	Europe / Netherlands /	Human	1	196997	33.02	Swab sample	IIb B.1

			North-Holland / Amsterdam						
611	hMpxV/Netherlands/NH-AUMC-0032/2022	EPI_ISL_15641570	Europe / Netherlands / North-Holland / Amsterdam	Human	1	196999	33.02	Swab sample	IIb B.1.14
612	hMpxV/Netherlands/NH-AUMC-0033/2022	EPI_ISL_15641571	Europe / Netherlands / North-Holland / Amsterdam	Human	1	196997	33.02	Swab sample	IIb B.1
613	hMpxV/Netherlands/NH-AUMC-0034/2022	EPI_ISL_15641572	Europe / Netherlands / North-Holland / Amsterdam	Human	1	196995	33.02	Swab sample	IIb B.1.7
614	hMpxV/Netherlands/NH-AUMC-0036/2022	EPI_ISL_15641574	Europe / Netherlands / North-Holland / Amsterdam	Human	1	196997	33.02	Swab sample	IIb B.1
615	hMpxV/Netherlands/NH-AUMC-0041/2022	EPI_ISL_15641579	Europe / Netherlands / North-Holland / Amsterdam	Human	1	197090	33.01	Swab sample	IIb B.1
616	hMpxV/Netherlands/NH-AUMC-0045/2022	EPI_ISL_15641583	Europe / Netherlands / North-Holland / Amsterdam	Human	1	197079	33.01	Swab sample	IIb B.1

617	hMpxV/Netherlands/NH-AUMC-0046/2022	EPI_ISL_156 41584	Europe / Netherlands / North-Holland / Amsterdam	Human	1	197080	33.01	Swab sample	IIb B.1
618	hMpxV/Netherlands/NH-AUMC-0048/2022	EPI_ISL_156 41586	Europe / Netherlands / North-Holland / Amsterdam	Human	1	197087	33.01	Swab sample	IIb B.1
619	hMpxV/Netherlands/NH-AUMC-0050/2022	EPI_ISL_156 41588	Europe / Netherlands / North-Holland / Amsterdam	Human	1	197087	33.01	Swab sample	IIb B.1
620	hMpxV/Netherlands/NH-AUMC-0051/2022	EPI_ISL_156 41589	Europe / Netherlands / North-Holland / Amsterdam	Human	1	197090	33.01	Swab sample	IIb B.1.2
621	hMpxV/Netherlands/NH-AUMC-0052/2022	EPI_ISL_156 41590	Europe / Netherlands / North-Holland / Amsterdam	Human	1	197088	33.01	Swab sample	IIb B.1
622	hMpxV/Netherlands/NH-AUMC-0053/2022	EPI_ISL_156 41591	Europe / Netherlands / North-Holland / Amsterdam	Human	1	197077	33.01	Swab sample	IIb B.1
623	hMpxV/Netherlands/NH-AUMC-0057/2022	EPI_ISL_156 41595	Europe / Netherlands /	Human	1	197077	33.01	Swab sample	IIb B.1

			North-Holland / Amsterdam						
624	hMpxV/Netherlands/NH-AUMC-0067/2022	EPI_ISL_156 41601	Europe / Netherlands / North-Holland / Amsterdam	Human	1	197074	33.01	Swab sample	IIb B.1
625	hMpxV/Germany/HE-HU-02/2022	EPI_ISL_157 04688	Europe / Germany / Hesse / Frankfurt	Human	1	197338	32.99	Not Available	IIb B.1.1
626	hMpxV/Germany/HE-HU-05/2022	EPI_ISL_157 05315	Europe / Germany / Hesse / Frankfurt	Human	1	197339	32.99	Not Available	IIb B.1
627	hMpxV/Germany/HE-HU-08/2022	EPI_ISL_157 05771	Europe / Germany / Hesse / Frankfurt	Human	1	197338	32.99	Not Available	IIb B.1
628	hMpxV/Ireland/L-NVRL-Z22IRL00254/2022	EPI_ISL_157 63814	Europe / Ireland / Limerick	Human	1	197099	33.01	Not Available	IIb B.1

Table S2: A List of Core Genes with Assigned Functions.

S.No.	Core Gene	FUNCTION
1	95019_hypothetical_protein	Putative monoglyceride lipase
2	95020_protein_K7	Bcl-2-like protein
3	95021_protein_F1	Caspase-9 inhibitor
4	95022_DUT	dUTPase
5	95023_Kelch_repeat_protein	kelch-like protein
6	95024_Ribonucleoside-dipho	Ribonucleotide reductase small subunit
7	95026_hypothetical_protein	CPXV053 protein
8	95027_hypothetical_protein	CPXV054 protein
9	95028_hypothetical_protein	Cytoplasmic protein
10	95029_Protein_F9	IMV membrane protein L1R
11	95030_VPK2	Serine/threonine-protein kinase
12	95031_hypothetical_protein	Protein F11
13	95032_Protein_F12	EEV maturation protein
14	95034_hypothetical_protein	Protein F14 (1)
15	95035_hypothetical_protein	Cytochrome C oxidase
16	95036_hypothetical_protein	Protein F15
17	95037_hypothetical_protein	Protein F16 (1)
18	95038_phosphoprotein_F17	DNA-binding phosphoprotein (1)
19	95039_CRMB	CRMB protein
20	95040_Protein_E2	Iev morphogenesis protein
21	95041_Protein_E3	Double-stranded RNA binding protein
22	95042_hypothetical_protein	DNA-directed RNA polymerase 30 kDa polypeptide
23	95044_Protein_E6	IMV membrane protein E6
24	95045_Protein_E7	Myristoylated protein E7
25	95046_Protein_E8	Membrane protein E8
26	95047_POL	Chain A, DNA polymerase
27	95048_Putative_FAD-linked_	Sulfhydryl oxidase
28	95049_Core_Protein_E11	Virion core protein E11
29	95050_hypothetical_protein	Iev morphogenesis protein
30	95051_Glutaredoxin-1	Glutaredoxin-1
31	95052_I1L	Telomere-binding protein I1
32	95053_I2L	IMV membrane protein I2
33	95054_Protein_I3	DNA-binding phosphoprotein (2)
34	95055_I4L	Ribonucleoside-diphosphate reductase (2)
35	95056_Telomere-binding_protein	Telomere-binding protein
36	95057_I7L	Viral core cysteine proteinase
37	95058_NPH2	RNA helicase NPH-II (2)
38	95059_Metalloendopeptidase	Metalloendopeptidase
39	95060_Protein_G3	Entry/fusion complex component
40	95062_Glutaredoxin-2	Glutaredoxin-2
41	95063_Putative_nuclease_G5	FEN1-like nuclease
42	95064_RPO7	DNA-directed RNA polymerase 7 kDa subunit
43	95065_hypothetical_protein	Nlpc/p60 superfamily protein
44	95066_Assembly_protein_G7	assembly protein G7
45	95067_VLTF1	Late transcription factor VLTF-1
46	95068_Myristoylated_protein	Myristylated protein

47	95069_L1R	IMV membrane protein L1R
48	95070_Protein_L2	Crescent membrane and immature virion formation protein
49	95072_L4R	Nucleic acid binding protein VP8/L4R
50	95073_Protein_L5	Membrane protein CL5
51	95075_TK	Thymidine kinase
52	95079_hypothetical_protein	DNA-directed RNA polymerase subunit 1
53	95080_H1L	Tyr/ser protein phosphatase
54	95081_Protein_H2	putative viral membrane protein [Variola virus]
55	95082_H3L	IMV heparin binding surface protein
56	95084_H5R	Late transcription factor VLTF-4 (1)
57	95085_TOP1	DNA topoisomerase type I
58	95086_Late_protein_H7	Late protein H7
59	95087_mRNA-capping_enzyme	mRNA capping enzyme large subunit
60	95088_D2L	Virion protein D2
61	95089_Core_protein_D3	Virion core protein D3
62	95090_UNG	Uracil DNA glycosylase superfamily
63	95091_Primase_D5	NTPase (1)
64	95092_VETFS	early transcription factor 70 kDa subunit
65	95093_DNA-directed RNA Pol	RNA polymerase subunit RPO18
66	95094_EL8	Carbonic anhydrase
67	95095_mRNA decapping protein	NUDIX domain protein
68	95096_mRNA decapping protein	MutT motif protein
69	95097_NPH1	Nucleoside triphosphatase I
70	95099_Scaffolding protein_D13	Rifampicin resistance protein
71	95100_VLTF2	late transcription factor VLTF-2 [Variola virus]
72	95101_VLTF3	Late transcription factor VLTF-3 (1)
73	95102_protein_A2.5	S-S bond formation pathway protein
74	95103_Major_Core_protein_4	Virion core protein P4b
75	95104_A4L	A5L protein-like
76	95105_DNA directed RNA Pol	DNA-directed RNA polymerase 19 kDa subunit
77	95107_VETFL	Early transcription factor 82 kDa subunit
78	95108_VITF3S	Intermediate transcription factor VITF-3 (1)
79	95110_Major_Core_protein_4	major core protein 4a precursor
80	95111_protein_A11	Viral membrane formation protein
81	95113_Virion_membrane_prot	virion membrane protein A13
82	95115_Core_protein_A15	Core protein A15
83	95116_Virion_membrane_prot	Myristylated protein
84	95117_Virion_membrane_prot.	IMV membrane protein P21
85	95118_Transcript_terminati.	DNA helicase
86	95119_Protein_A19	Zinc finger-like protein (1)
87	95120_Virion_membrane_prot.	IMV membrane protein A21
88	95121_DNA_polymerase_proce.	DNA polymerase processivity factor
89	95122_Resolvase_A22	Holliday junction resolvase
90	95123_VITF3L	Intermediate transcription factor VITF-3 (2)
91	95129_A-type_inclusion_pro.	A-type inclusion body protein

92	95130_Protein_A26	MPXV-WRAIR131
93	95132_Envelope_protein_A28	Envelope protein A28 homolog
94	95133_DNA-directed_RNA_pol.	DNA-directed RNA polymerase 35 kDa subunit
95	95134_Protein_A30	IMV membrane protein A30
96	95135_hypothetical_protein	A32.5L
97	95136_hypothetical_protein	CPXV166 protein
98	95137_Protein_A32	ATPase A32
99	95138_Protein_A33	EEV glycoprotein (1)
100	95139_Protein_A34	EEV glycoprotein (2)
101	95140_Protein_A35	MHC class II antigen presentation inhibitor
102	95143_Hypothetical-Protein	CD47-like protein
103	95144_Protein_A41	Chemokine binding protein
104	95145_Profilin	Profilin domain protein
105	95146_Protein_A43	Type-I membrane glycoprotein
106	95147_hypothetical_protein	MPXV_gp150
107	95149_hypothetical_protein	Copper/zinc superoxide dismutase
108	95152_TMK	Thymidylate kinase
109	95153_LIG	DNA ligase (2)
110	95154_hypothetical_protein	M137R
111	95157_HA.faa	Hemagglutinin
112	95159_VPK1	Ser/thr kinase
113	95160_B4R	MPXV-WRAIR157
114	95162_PS-HR	EEV type-I membrane glycoprotein
115	95164_hypothetical_protein	Virulence protein
116	95165_Soluble_interferon_g	Soluble interferon-gamma receptor-like protein
117	95169_putative_serine-thre	Ser/thr kinase
118	95170_SPI-2.	Serpin

Table S3: List of Core Genes with Position-Specific Mutations in a Specified Number of Isolates, Displaying the dn/ds Ratios and Associated p-Values as Determined by Codeml Analysis.

S. No.	Protein No.	Name of the protein	Amino acid in Reference	Mutated amino acid	Position	Type of mutation	No. of strains with similar mutation	ωvalue (Codeml)	p-value
1	95130	Protein A26	T	M	14		600	0.3179	0.0277
			A	E	21		2		
			R	H	205		6		
			A	T	343		627		
			T	I	355		601		
			Y	H	358		602		
			D	E	366		626		
			D	K	366		1		
			D	I	370		600		
			D	I	371		601		
			D	I	372		9		
			D	I	373		28		
			D	I	374		19		
			Ref sequence differs from the others	Also a few differ from still others	376-509				
2	95132	Envelope protein A28	R	I	101		1	0.3425	0.015
3	95133	DNA directed RNA Polymerase	Y	S	42		27	0.3159	0.022
			T	A	222		27		
			Long stretch different in some strains only		191-323		5		
			I	M	261		27		
			S	F	243		3		
4	95134	Protein A30	T	M	71		1	0.3164	0.0152
			M	V	24		27		
			One aa deletion at position 61	Q a.a	61		27		
5	95135	Hypothetical Protein	NO MUTATIONS					0.3248	0.0497
6	95136	Hypothetical Protein	T	A	2		27	0.2554	0.0021
			L	S	36		27		
			3 aa insertion in the sequences other than reference	NYN a.a	124-126		621		
7	95137	Protein A32	NO MUTATIONS					0.3022	0.0154
8	95138	Protein A33	One aa deletion at position 2	M a.a	2		627	0.3928	0.0096
			E at position 67 in reference	K at position 66 in other strains	66/67		28		
9	95139	Protein A34	NO MUTATIONS					0.3391	0.383

**19**	**95140**	**Protein A35**	N	D	38		1	**0.3183**	**0.0056**
			I	A	46		27		
			I	V	46		601		
**11**	**95143**	**Hypothetical Protein**	NO MUTATIONS						
**12**	**95144**	**Protein A41**	D	G	23		600	**0.3213**	**0.00306**
			M	I	103		28	**0.3378**	**0.00182**
			H	Y	108		5		
			G	R	181		1		
			S	F	153		1		
			D	N	178		1		
**13**	**95145**	**Profilin**	L	F	61		4	**0.391**	**0.0076**
**14**	**95146**	**Protein A43**	2 a.a deletion at position 1/2/3	MM a.a	1or2or3		11	**0.2751**	**0.00335**
			1 a.a addition at position 1/2/3	M a.a	1or2or3		9		
			1 a.a deletion at position 1/2/3	M a.a	1or2or3		4		
			3 a.a addition at position 1/2/3	MMM a.a	1or2or3		1		
			E	V	56		1		
			V	D	167		628		
			Y	D	160		28		
			A	V	125		28		
			T	M	145		600		
			A	T	125		2		
**15**	**95147**	**Hypothetical Protein**	1 a.a addition at position 34	E	34		1	**0.3102**	**0.00383**
			V	I	29		14		
			A	T	30		627		
			E	D	57		1		
**16**	**95149**	**Hypothetical Protein**	G	V	28		3	**0.3172**	**0.00138**
**17**	**95152**	**TMK**	G	E	98		1	**0.3595**	**0.00525**
			I	V	139		28		
**18**	**95153**	**LIG**	A	T	173		2	**0.3316**	**0.00301**
			S	F	138		1		
			T	S	330		1		
			D	N	325		1		
			D	N	442		6		
			5 a.a deletion at position 555 to 559	LNSYI a.a	555 - 559		28		
**19**	**95154**	**Hypothetical Protein**	L	M	85		27	**0.3432**	**0.0076**
			P	S	95		2		
			S	Y	127		27		
			A	V	132		1		
			A	T	107		5		
**20**	**95157**	**HA**	A	T	167		602	**0.3472**	**0.00632**
			E	D	177		29		

			D	N	178		1		
			I	T	205		27		
			E	K	121		14		
			A	V	243		1		
			S	L	288		8		
**21**	**95158**	**Hypothetical Protein**	NO MUTATIONS					**0.3185**	**0.00071**
**22**	**95159**	**VPK1**	E	K	289		1	**0.3433**	**0.0048**
			4 a.a deletion at position 300-303	KNFC a.a	300-303		27		
**23**	**95160**	**B4R**	2 a.a deletion at position 1-2	MA a.a	1 and 2		27	**0.3922**	**0.005**
			H	Y	122		6		
			D	N	174		1		
			E	K	226		1		
			G	S	351		1		
			S	R	421		27		
			P	S	326		3		
			R	K	447		1		
**24**	**95162**	**PS-HR**	H	Y	53		1	**0.3596**	**0.0098**
			G	R	115		2		
			G	S	103		3		
			S	L	50		1		
			E	K	84		1		
			G	E	105		1		
			P	S	221		6		
**25**	**95164**	**Hypothetical Protein**	G	E	100		27	**0.2875**	**0.0087**
**26**	**95165**	**Soluble interferon-gamma receptor-like protein**	R	I	108		600	**0**	**0.001**
			L	F	263		589		
			S	A	264		1		
			I	F	265		1		
			D	T	267		1		
			3 a.a addition at position 268	KYS a.a	268		1		
**27**	**95169**	**Putative ser/thr protein kinase**	D	N	71		600	**0.3182**	**0.0098**
**28**	**95170**	**SPI-2**	R	Q	133		600	**0.3653**	**0.00524**
			I	V	100		27		
			L	M	87		2		
			D	N	94		1		
			E	K	174		1		
			E	K	250		6		
			S	T	179		4		
			H	Y	169		1		
**29**	**95019**	**Putative monoglyceride lipase**	T	A	3		27	**0.3599**	**0.00989**
			S	F	39		1		
			E	K	44		1		

**30**	**95020**	**Bcl-2 like protein**	A	V	63		600	**0.3358**	**0.00723**
			T	A	109		27		
			D	N	134		2		
			M	L	148		27		
**31**	**95021**	**Protein F1**	D	E	15		10	**0.3286**	**0.00987**
**32**	**95022**	**DUT**	D	S	88		608	**0.3819**	**0.00231**
**33**	**95023**	**Kelch repeat protein**	R	C	48		585	**0.3409**	**0.00555**
			I	K	166		627		
			D	N	187		6		
			N	D	229		627		
			K	I	232		600		
			H	Y	269		1		
			R	K	353		1		
			P	S	436		1		
			I	V	464		1		
			I	G	476		2		
			D	N	478		29		
			N	G	481		24		
			E	D	483		6		
			M	K	485		3		
			E	V	486		19		
			W	N	487		19		
			G	F	489		19		
			K	E	490		19		
			-	I	491		19		
			-	K	492		19		
**34**	**95024**	**Ribonucleotide reductase small subunit**	NO MUTATIONS					**0.3541**	**0.00696**
**35**	**95026**	**Hypothetical Protein**	L	F	72		600	**0.2935**	**0.00325**
			Q	T	73		600		
			-	I	74		600		
			-	N	75		600		
**36**	**95027**	**Hypothetical Protein**	NO MUTATIONS					**0.3468**	**0.00151**
**37**	**95028**	**Hypothetical Protein**	S	T	10		1	**0.3856**	**0.00044**
			R	C	11		1		
			R	C	20		1		
			A	T	31		4		
**38**	**95029**	**Protein F9**	P	S	78		585	**0.3431**	**0.00296**
			G	E	38		6		
			E	K	87		1		
			S	L	128		2		
			P	S	130		2		
			F	C	63		2		
**39**	**95030**	**VPK2**	Q	R	12		1	**0.306**	**0.00151**
			E	K	10		1		
			E	K	199		1		
			G	R	438		600		

			T	A	402		1		
**40**	**95031**	**Hypothetical Protein**	T	A	118		627	**0.3559**	**0.0071**
			A	V	142		2		
			S	L	38		2		
			D	N	102		1		
			L	I	347		1		
			S	L	156		7		
			A	T	227		5		
			D	N	273		1		
			S	F	164		1		
**41**	**95032**	**Protein F12**	E	K	125		585	**0.3355**	**0.0068**
			S	N	151		628		
			R	C	33		11		
			R	C	38		1		
			V	D	218		627		
			C	Y	203		1		
			A	V	323		600		
			T	N	602		6		
			C	Y	622		27		
			S	F	609		1		
**42**	**95034**	**Hypothetical Protein**	D	N	62		1	**0.3242**	**0.00427**
**43**	**95036**	**Hypothetical Protein**	N	S	119		1	**0.2826**	**0.0091**
**44**	**95037**	**Hypothetical Protein**	D	E	129		2	**0.3074**	**0.00892**
			P	S	110		4		
**45**	**95038**	**Hypothetical Protein**	M	I	79		4	**0.4124**	**0.0424**
**46**	**95039**	**CRMB**	I	L	26		26	**0.8153**	**0.0078**
		Special note: on comparison with reference genome the protein of strain 156_13953610; 99% match seen, however matching starts with query from amino acid 1 and subject with amino acid 8.							
**47**	**95040**	**Protein E2**	A	S	88		27	**0.3084**	**0.0043**
			A	T	88		1		
			H	Y	40		1		
			E	K	387		1		
			P	H	348		2		
			E	K	430		1		
			R	K	560		1		
			R	H	457		1		
			G	A	684		6		
			E	K	658		1		
			S	L	655		1		
**48**	**95041**	**Protein E3**	R	K	120		1	**0.3791**	**0.00128**
			A	T	17		27		
			N	D	23		27		
			H	N	61		27		
**49**	**95042**	**Hypothetical Protein**	K	R	83		27	**0.3502**	**0.00147**
			H	N	97		27		

			E	V	179		1		
**50**	**95044**	**Protein E6**	P	S	251		27	**0.3127**	**0.00315**
			D	E	341		27		
			E	K	542		1		
			E	K	493		1		
**51**	**95045**	**Protein E7**	D	N	121		4	**0.3071**	**0.0016**
			D	N	49		1		
			C	R	84		1		
			E	G	42		1		
			N	Y	43		1		
			N	Y	44		1		
			E	K	17		2		
			S	G	144		1		
**52**	**95046**	**Protein E8**	V	A	91		1	**0.3382**	**0.0027**
			H	Y	205		27		
**53**	**95047**	**POL**	L	F	108		585	**0.3443**	**0.0013**
			T	I	71		1		
			R	C	25		1		
			D	N	143		1		
			W	L	411		600		
			I	T	428		27		
			A	S	484		27		
			I	V	501		27		
			V	L	573		1		
			S	L	736		4		
			N	D	785		6		
			E	K	888		1		
**54**	**95048**	**Putative FAD-linked**	D	N	56		585	**1.3685**	**0.0085**
			V	I	10		27		
			L	I	34		5		
**55**	**95049**	**Core protein E11**	NO MUTATIONS					**0.3229**	**0.003**
**56**	**95050**	**Hypothetical Protein**	P	L	43		1	**0.3249**	**0.0067**
			S	L	17		1		
			S	L	13		1		
			R	C	180		1		
			N	D	199		27		
			V	I	441		601		
			F	S	459		627		
			I	T	514		1		
			L	P	515		2		
			F	S	516		2		
			N	K	517		2		
			K	E	523		1		
			L	R	524		1		
			R	C	665		16		
			V	S	653		1		
			I	F	654		1		

			I	E	656		1		
**57**	**95051**	**Glutaredoxin-1**	E	K	3		2	**0.3458**	**0.00435**
			R	K	72		1		
**58**	**95052**	**I1L**	E	K	243		1	**0.3281**	**0.0005**
**59**	**95090**	**UNG**	D	N	73		1	**0.364**	**0.00274**
			D	E	192		3		
**60**	**95091**	**Primase D5**	S	L	21		1	**0.3413**	**0.00622**
			D	N	263		1		
			D	N	729		4		
			P	S	785		1		
**61**	**95092**	**VETFS**	G	E	4		2	**0.3156**	**0.00102**
			G	K	4		4		
			R	K	256		602		
			V	A	281		3		
			D	G	381		3		
			S	N	413		599		
			A	Y	462		600		
			D	N	463		1		
			Y	F	477		1		
			E	K	606		2		
**62**	**95093**	**DNA directed RNA polymerase**	V	I	31		1	**0.3685**	**0.00568**
			E	A	55		1		
			T	R	88		6		
**63**	**95094**	**E8L**	A	T	19		27	**0.9333**	**0.001**
			H	R	213		6		
			V	A	261		626		
**64**	**95095**	**mRNA-decapping protein**	Deletion and shortening of 4 bases				4	**0.2911**	**0.0032**
			S	L	189		2		
**65**	**95096**	**mRNA-decapping protein**	D	N	58		1	**0.3064**	**0.0011**
			H	Y	198		1		
**66**	**95097**	**NPH1**	A	V	29		626	**0.3396**	**0.00215**
			D	N	155		1		
			I	T	172		27		
			A	T	313		29		
			E	K	344		1		
			C	Y	436		28		
			E	K	511		1		
**67**	**95099**	**Scaffold Protein D13**	N	H	97		626	**0.346**	**0.326**
			E	D	111		27		
			I	L	399		626		
			V	I	514		599		
**68**	**95100**	**VLTF2**	NO MUTATIONS					**0.3421**	**0.0019**
**69**	**95101**	**VLTF3**	H	Y	193		3	**0.2932**	**0.00178**
			M	I	194		1		
			S	F	196		1		
**70**	**95102**	**Protein A2.5**	NO MUTATIONS					**0.3247**	**0.00214**

**71**	**95103**	**Major core protein**	A	S	460		1	**0.3659**	**0.00162**
			K	Q	593		3		
			Q	R	594		3		
**72**	**95104**	**A4L**	A	T	63		599	**0.426**	**0.00125**
			A	V	122		27		
			S	F	121		1		
			T	I	107		1		
			L	I	270		27		
			S	L	175		1		
**73**	**95105**	**DNA directed RNA polymerase**	NO MUTATIONS					**0.352**	**0.0043**
**74**	**95107**	**VETFL**	R	Q	533		1	**0.3141**	**0.0064**
			F	S	594		27		
			D	N	592		2		
			G	E	619		1		
**75**	**95108**	**VITF3S**		K	39		1	**0.3311**	**0.0016**
			V	A	43		626		
			E	D	226		27		
			-	YNL	293-295	Addition of 3 Nucleotides	1		
**76**	**95110**	**Major Core Protein 4**	2 variants (4 proteins different from remaining)					**0.3438**	**0.00373**
			D	N	98		590		
**77**	**95111**	**Protein A11**	E	K	213		1	**0.3437**	**0.00341**
			D	N	214		7		
			A	V	257		1		
**78**	**95113**	**Virion membrane protein**	A	T	17		588	**0.3479**	**0.00251**
			S	F	40		1		
**79**	**95115**	**Core protein A15**	NO MUTATIONS					**0.3193**	**0.00325**
**80**	**95116**	**Virion Membrane Protein**	NO MUTATIONS					**0.3359**	**0.00219**
**81**	**95117**	**Virion Membrane Protein**	2 variants				611	**0.3285**	**0.00786**
**82**	**95118**	**Transcript termination protein**	D	N	29		5	**0.3397**	**0.0012**
			E	K	62		585		
			R	Q	243		580		
			T	I	264		585		
			ONE MORE VARIANT (INDELS)				5		
			P	A	348		585		
			E	K	435		580		
**83**	**95119**	**Protein A19**	S	N	3		1	**0.4027**	**0.0057**
			G	A	9		1		
			M	I	10		1		
			K	S	11		1		
			A	V	76		2		
**84**	**95120**	**Virion Membrane Protein**	M	V	39		627	**0.3518**	**0.00023**
			D	N	85		2		
**85**	**95121**	**DNA polymerase processivity factor**	Y	S	75		1	**0.31**	**0.00888**

			E	K	109		1		
**86**	**95122**	**Resolvase A22**	D	N	46		1	**0.3508**	**0.0056**
			S	L	97		1		
**87**	**95123**	**VITF3L**	S	L	43		1	**0.3108**	**0.008**
			D	N	100		30		
			S	L	307		30		
**88**	**95129**	**A type inclusion protein**	R	Q	530		1	**0.3693**	**0.0017**
			R	Q	547		1		
			R	C	564		1		
			T	D	583		27		
**89**	**95053**	**I2L**	NO MUTATIONS					**0.2877**	**0.0096**
**90**	**95054**	**Protein I3**	C	Y	45		27	**0.3546**	**0.0066**
**91**	**95055**	**14L**	E	V	195		1	**0.3723**	**0.00313**
			I	C	255		1		
**92**	**95058**	**NPH2**	S	P	391		627	**0.3206**	**0.00772**
			R	C	620		598		
**93**	**95059**	**Metalloendopeptidase**					11 variants	**0.3181**	**0.0064**
			T	A	328		18		
			L	K,N	552		8		
			M	T	519		11		
			M	I	519		1		
			E	K	415		6		
			D	Y	361		1		
**94**	**95062**	**Glutaredoxin-2**	A	T	22		3	**0.3655**	**0.0373**
			E	K	29		1		
**95**	**95063**	**Putative Nuclease G5**	A	E	178		627	**0.622**	**0.00128**
			D	E	303		25		
			E	K	109		1		
**96**	**95065**	**Hypothetical Protein**	V	L	96		627	**0.3008**	**0.0034**
			V	I	62		10		
			S	N	73		10		
			D	N	143		1		
**97**	**95066**	**Assembly protein G7**					3 variants	**0.3325**	**0.00172**
			C	K	20		1		
			D	N	196		596		
			I	N	244		24		
**98**	**95067**	**VLTF1**	S	L	30		585	**0.345**	**0.00325**
			D	N	88		585		
**99**	**95068**	**Myristoylated protein**	M	I	142		575	**0.3706**	**0.0096**
			P	H	19		1		
			D	N	54		2		
			D	N	141		2		
			T	R	175		29		
			L	H	194		70		
			S	L	213		1		
**100**	**95073**	**Protein L5**	V	L	103		1	**1.52033**	**0.00315**

			D	N	124		5		
**101**	**95075**	**Thymidine kinase**	T	A	153		27	**0.3581**	**0.00214**
**102**	**95089**	**Core Protein D3**	E	K	47		8		
			H	Y	42		3		
			V	I	105		2		
**103**	**95087**	**mRNA capping enzyme**	T	A	3		627	**0.3317**	**0.00305**
			E	K	700		13		
**104**	**95085**	**TOP1**	I	V	77		27	**0.3111**	**0.006**
			N	S	194		627		
**105**	**95086**	**Late Protein H7**	Multiple mutations		2-146		626 variants	**0.3472**	**0.0078**
**106**	**95080**	**H1L**	I	V	80		600	**0.3256**	**0.001**
			I	E	80		27		
			S	F	92		4		
			S	E	92		27		
			T	A	135		600		
			T	N	135		27		
**107**	**95081**	**Protein H2**	E	K	153		627	**0.3579**	**0.008**
